# Validation of an automated system for aliquoting of HIV-1 Env-pseudotyped virus stocks

**DOI:** 10.1371/journal.pone.0190669

**Published:** 2018-01-04

**Authors:** Anke Schultz, Anja Germann, Martina Fuss, Marcella Sarzotti-Kelsoe, Daniel A. Ozaki, David C. Montefiori, Heiko Zimmermann, Hagen von Briesen

**Affiliations:** 1 Fraunhofer Institute for Biomedical Engineering IBMT, Joseph-von-Fraunhofer-Weg 1, Sulzbach, Germany; 2 Department of Surgery, Duke University Medical Center, Durham, North Carolina, United States of America; 3 Molecular and Cellular Biotechnology/Nanotechnology, Saarland University, Saarbrücken, Germany; 4 Marine Sciences Universidad Católica del Norte, Antafogasta/Coquimbo, Chile; George Mason University, UNITED STATES

## Abstract

The standardized assessments of HIV-specific immune responses are of main interest in the preclinical and clinical stage of HIV-1 vaccine development. In this regard, HIV-1 Env-pseudotyped viruses play a central role for the evaluation of neutralizing antibody profiles and are produced according to Good Clinical Laboratory Practice- (GCLP-) compliant manual and automated procedures. To further improve and complete the automated production cycle an automated system for aliquoting HIV-1 pseudovirus stocks has been implemented. The automation platform consists of a modified Tecan-based system including a robot platform for handling racks containing 48 cryovials, a Decapper, a tubing pump and a safety device consisting of ultrasound sensors for online liquid level detection of each individual cryovial. With the aim to aliquot the HIV-1 pseudoviruses in an automated manner under GCLP-compliant conditions a validation plan was developed where the acceptance criteria—accuracy, precision as well as the specificity and robustness—were defined and summarized. By passing the validation experiments described in this article the automated system for aliquoting has been successfully validated. This allows the standardized and operator independent distribution of small-scale and bulk amounts of HIV-1 pseudovirus stocks with a precise and reproducible outcome to support upcoming clinical vaccine trials.

## Introduction

The HIV-1 Env-pseudotyped viruses are a widely used test reagent in neutralization assays for the assessment of neutralizing antibody responses elicited from candidate vaccine within clinical trials. They are displaying, as predominantly replication incompetent viruses [1] capable of a single round infection, a specific spectrum of genetically, antigenically and geographically diverse envelope proteins on their surface. Additional beneficial aspects are the clonal character of the viruses with precisely known env sequences mediating a high degree of stability and reproducibility regarding the outcome of the assay results, which is of main importance in the field of vaccine development for clinical studies [2]. HIV-1 pseudoviruses are further of great interest when trying to identify potential new target sites for vaccine design, e.g. high-mannose glycan-dependent epitopes to induce a broad and potent neutralizing antibody response [3]. Next to the preventive arm of an HIV-1 vaccine, the research moved also in another direction, namely the characterization of broadly neutralizing antibodies as therapeutic tools for the treatment of HIV-1 infected individuals, classified by assaying pseudoviruses as test reagents in TZM-bl neutralization experiments [4]. Even the latest discovery of the HIV-1 antibody 3BNC117 as suppressor of viral rebound from the latent reservoirs during analytical treatment interruption applies pseudoviruses to determine the sensitivity of the rebound viruses to 3BNC117 [5].

However, the HIV-1 pseudotyped viruses are originated, as biological reagents, from cell culture systems by processes that carry certain variances due to the complex biology behind them, which may be influenced by the quality of the starting material (e.g. the plasmid DNA), transfection reagent and cell culture conditions [6], but also variabilities in the assay performance. The implementation of standardized methods and reagents, the integration of a quality assurance system and validation of processes, in addition to an automated system for the large-scale preparation of the pseudoviruses, allowed precise outcomes with the further benefit to be completely independent from external influences and operator specific variations during the preparation performance [7], a crucial asset if the handled compounds contribute to vaccine trials. By validation of such an automated system two aspects have to be covered, the automated system itself and its associated components as well as the preservation of the uniformity regarding the biological product. For the automation, the ISO- (The International Organization of Standardization; http://www.iso.org) and GMP- (Good Manufacturing Practice) standards (http://ec.europa.eu/health/documents/eudralex/vol-4/index_en.htm) [8], are commonly used in the pharmaceutical industry or for the manufacturing of medical products. As the pseudoviruses are key reagents in clinical trial testing, the specifications for Good Clinical Practice (GCP) and Good Laboratory Practice (GLP) should also be taken into account [9,10]. More appropriately, Good Clinical Laboratory Practice (GCLP) Guidelines were successfully used for the validation of the cell-based TZM-bl- neutralizing antibody assay, which utilizes pseudoviruses as test reagents as well as for the implementation of quality control programs [11–15]. These guidelines include i.a. the organization, the qualification of the personnel and equipment, the performance, the adequate documentation, the creation of Standard Operating Procedures (SOPs) as well as the very important quality control of the product before its release [11]. Therefore, for validation of such a combination of automation and biological substance the GCLP guidelines are the most suitable ones.

This article describes the effort to further improve the standardization of the HIV-1 pseudovirus production procedure by implementing an automated system for aliquoting of HIV-1 pseudovirus stocks up to liter-scale. Next to the principal technical feasibility to handle the racks containing 48 cryovials, the simultaneous de- and recapping of the vials and distribution of 1 ml of the virus stock under sterile conditions, a high degree of traceability by liquid level detection via ultrasound sensors, the online monitoring of the complete process and the related automated documentation were of particular interest. However, the main focus was set on the validation of the automated platform and the aliquoting process according to GCLP guidelines in consideration of the selected validation parameters such as accuracy, precision, specificity and robustness, because this is the fundamental requirement for the intended application of the pseudoviruses in upcoming clinical studies in HIV vaccine research.

## Material and methods

### Cell lines

The cell line 293T/17 was used for the preparation for the HIV-1 Env-pseudotyped viruses and was obtained through LGC Promochem (ATCC^®^ CRL-11268^™^) and the TZM-bl cells for the titration assay and neutralization assay were received from NIH AIDS Research and Reference Reagent Program (ARRRP, catalog no. 8129). Both cell lines were cultivated as previously described [7,13,15].

### Virus preparation, cryopreservation and titration

The HIV-1 pseudovirus stocks were produced according to the manual procedure as well as using the automated system for virus production. The protocol for both procedures have been published previously [7,13,15]. With regard to the aliquotation process, the manual aliquoting procedure was specified as reference method within the validation study and occurred in 2.0 ml cryovials (Sarstedt). The manually aliquoted samples were transferred into a carton cryobox of 135x135x50mm in size (RatioLab) containing in total 64 vials and frozen at -80°C in a steel rack (MUT GmbH) with space for 16 cryoboxes. The automatically aliquoted 2.0 ml cryovials (Greiner Bio-One) were placed in a 48-way platic tube rack (FludiX), which was frozen completely in an entire compartment of the -80°C freezer following the automated aliquoting procedure. The titration assay to determine the virus dilution at a relative luminescence unit (RLU) of 150,000 occurred according to the method described before [7,13,15]. Within this study, the luminescence was measured using Victor X3 luminometer (Perkin Elmer).

### TZM-bl assay

The neutralization assay was performed by using TZM-bl cells formerly described by Montefiori [13,16] which is a modified version of the assay by Wei et al. [17] and determine the Inhibitory Concentration providing 50 value (IC50 = 50% reduction of the RLU values compared to the virus control; half maximal inhibitory concentration) by measuring the luminescence with the Victor X3 luminometer (Perkin Elmer). In order to control for lot-to-lot variations and virus stock integrity, two parallel neutralization assays were run with the automatically aliquoted HIV pseudovirus and a manually aliquoted reference virus of the same type by using a panel of five specified control reagents: sCD4 (Progenics), IgG1b12, 2F5, 4E10 and TriMab consisting of 2G12, IgG1b12 and 2F5 (Polymun) with an initial concentration of 25 μg/ml. The indicated concentration of the control reagents, which should lead to 50% neutralization, must agree within 3-fold between the automatically aliquoted virus stock and the reference virus for 80% of the tested reagents.

### Automated platform

**Fig 1:**
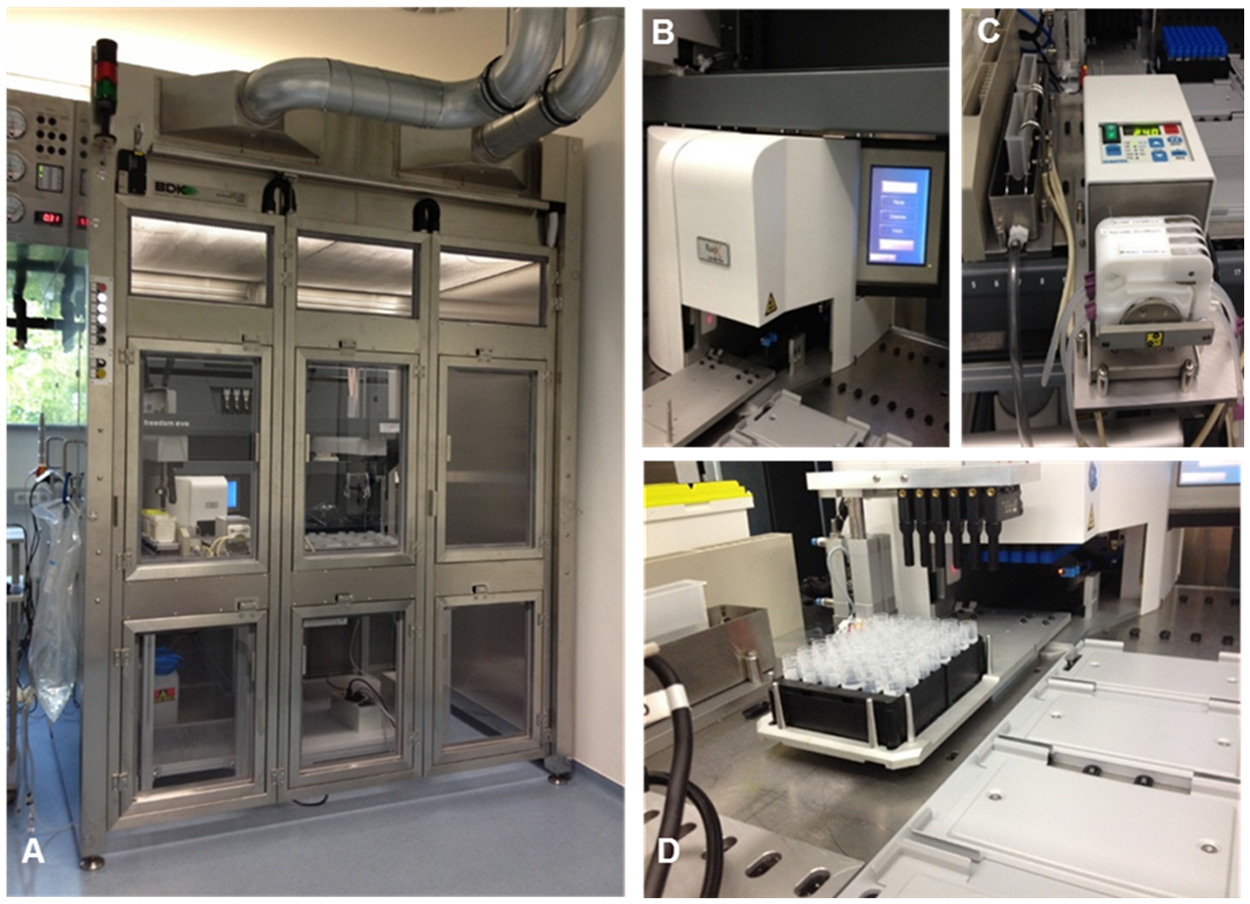
Automated system for aliquoting and the associated components. (A) Complete view of the system, (B) Decapper to open and close 48 cryovials simultaneously (C) Tubing pump mediating the transport of the virus containing solution from the virus supply bottle to the trough on the worktable, (D) at the back: ESD-system consisting of 6 ultrasound sensors integrated in the bridge for detecting the liquid level inside the tubes and the cap status; at the front: 48-way tube rack with 48 opened cryovials.

The automation platform for filling of HIV-1 pseudovirus stocks comprises of a modified Tecan-based Evo 150 system. It covers an area of 2.25 x 2.18 meters and includes a robot platform, a Decapper (XSD-48Pro, FluidX) and a tubing pump (Reglo Digital, Ismatec). In addition, the system is housed in a biosafety cabinet class II (Laminar Flow Hood Type WK 12.19-S3, BDK) to protect personnel, environment and the product (Fig 1A). The core of the automated aliquotation system is a modular standard Freedom EVO 150 worktable for processing and liquid handling and is able to fulfill the requirements for the automated de- and recapping of the 2.0 ml cryovials (Greiner Bio-one) as well as the filling of HIV-1 pseudovirus containing supernatant. It contains carriers for a maximum of 20 racks for 48 cryovials and includes a stainless steel holder with two troughs, which are used to provide the virus stock and the peracetic acid disinfection solution (e.g. 0.4% Peraclean). The 0.4% Peraclean (Altmann Analytik) solution is provided by a storage bottle underneath the worktable and is connected to the tubing system. However, usually sterile and distilled water (Lonza, Bulk Packed Sterile WFI) is the system liquid supplied to the tubing system via a 20 liter storage bag. The liquid handling arm (LiHa) is part of the liquid system and is used for pipetting tasks. It contains eight reusable steel channels, where the 1000 μl conductive disposable tips (DiTis, Tecan) are attached before the distribution of the virus containing supernatant. The liquids provided at the worktable (virus supernatant, disinfectant, water) and the system liquid are aspirated and dispensed with built-in 1 ml syringes. Another integrated hardware component is the centric gripper of the robotic manipulator arm (RoMa), which transfers the rack for 48 cryovials within the system. The racks can be moved from the carrier on the worktable to the transfer station of the Decapper and the other way around. The LiHa and the RoMa are controlled by the Tecan Freedom Evoware Standard 1.1 Software, which is in turn controlled by the Enhanced Safety Device (ESD)-software (Cellomation).

The Decapper (Fig 1B) is located on the worktable and is controlled via Freedom Evoware Standard 1.1 Software (Tecan) and thus with the ESD-Software as well. For decapping, a rack containing 48 closed 2.0 ml cryovials is transported to the rack transfer station by the RoMa. The rack is moved into the Decapper and all caps of the tubes are screwed simultaneously. In the following step the rack with the opened cryovials is moved out of the Decapper, whereas the caps stay inside the machine, until the recap process will be finalized. After filling of the virus containing solution in 2x 500 μl pipetting tasks the rack is returned inside the machine, all caps are replaced and screwed on the vials again.

The tubing pump Reglo Digital from Ismatec (Fig 1C) is a four channel tubing pump for application with a low flow rate (0.002–43 ml/min) and is controlled by the ESD-software respectively the Evoware Standard software (Tecan). For the described automated aliquoting system, the flow rate is set to 24 ml/min. The general function of the tubing pump is the transfer of the pseudovirus stock from the virus supply bottle to the trough on the worktable with one tube and the other way around with two tubes to prevent the accidental overflow of the virus containing solution.

The ESD-system was introduced to check and to monitor the cap status of the automated decapping and recapping process as well as to prevent an accidental collision of the DiTis with not correctly opened cryovials. The hardware components of the ESD consist of six ultrasound sensors (US) installed via a bridge in front of the Decapper (Fig 1D). By scanning each row of the 48-way tube rack during in and out movement of the Decapper transfer station, each individual cryovial inside the rack is analyzed. An additional function of the device is the detection of the liquid level inside each cryovial and the time related documentation in the Structured Query Language- (SQL) database. For an optimal performance of the US they are positioned in two different distances to the tube rack. Therefore, the US bridge is lowered and raised pneumatically by the ESD. In the lower position of the bridge the liquid level is detected and in the raised one the cap status. Another safety aspect of the ESD-system is the verification of the complete opening of the Decapper drawer for the following accurate distribution of the virus stock into the cryovials. The ESD-software consists of two programs, ESD.exe and ResultViewer.exe. The hardware of the ESD is controlled by a programmable logic controller (PLC) which is mounted in an electronic box. The PLC itself communicates with the Decapper and the peristaltic pump through their respective communication protocol through two serial interfaces (RS232). Communication of the PLC to the PC-ESD-software is realized by Ethernet [18].

### Validation design

The accuracy and precision of the automated method was tested with respect to the volume distributed by the eight channels and connected DiTis by gravimetric measurement (Tecan) with the selected volumes of 100 μl and 500 μl, which were dispensed into the prefilled vessel inside the weight chamber of the SAG 285 balance from Mettler Toledo. Within an EVOware script each channel and syringe was tested in twelve transfer steps for the volume of 100 μl and 24 transfers for the volume of 500 μl distilled water (LONZA, Bulk Packed Sterile WFI, density: 1.0 g/ml). Based on these weighing results, the ranges were set as follows: %Acc ≤5.0% and %CV ≤0.75% by calculating the accuracy as the deviation to the target volume divided by the target volume and the precision as the standard deviation (SD) divided by the average of all measurements (S1 and S2 Tables).

The accuracy and precision of the liquid level detection using the ultrasound sensors were determined by (I) a 10-times measurement of a standardized aluminium block resulting in the acceptance range of %Acc ≤5.0% and %CV ≤0.75%, (II) a 10-times measurement of one rack with 1000 μl of growth medium plus 20% FBS automatically aliquoted resulting in the acceptable limit of %Acc ≤5.0% and %CV ≤3.0% as well as limit for the detected volume between 950 μl and 1050 μl for both (S3 and S4 Tables).

The limit to test the precision of the automatically aliquoted HIV-1 Env-pseudotyped viruses was defined by aliquoting three racks of the pseudovirus stock ZM214M.PL15 and ten racks of the pseudovirus SF162.LS and their evaluation in the titration assays as well as in parallel TZM-bl tests. The titration data were used to set the range for automatically aliquoted pseudovirus to be within 3-fold of manually aliquoted virus stocks before the automated aliquoting process (Tables A and B in S5 Table). Based on this and historical data of the titration assays (Tables A and B in S6 Table) the acceptance criteria for the intra-assay variability was set to %CV ≤35.0%. The TZM-bl assay pass criterion for the validation experiments specified that the neutralization titers for at least 80% of the assayed reagents (sCD4, IgG1b12, 2F5, 4E10 and TriMab) must agree within 3-fold between the data from the automatically aliquoted pseudovirus and the manually aliquoted reference stock (S7 Table).

Robustness and specificity refer to the stability of the HIV pseudoviruses over the entire period of the automated aliquoting process (e.g. 1 h and 50 min for eleven racks). To test if there is no detrimental effect to the virus left at room temperature (RT) being unagitated, a large-scale virus stock (40 T-75 flasks) of the pseudovirus ZM249M.PL1 was produced and aliquoted manually after an incubation of 2 hrs and 20 min at RT. Based on parallel titration assays (Table 1) and on data of previous studies (S8 Table) the stability of the pseudovirus stocks at RT could be demonstrated up to an incubation time of 6 hrs. Furthermore, the titration data shown in Table 1 were used to establish the acceptance limit of 3-fold at the dilution of 150,000 RLU between the tested aliquots directly frozen and the one incubated at RT. The acceptance limits for the direct comparison of automatically versus manually aliquoted virus stocks correspond to the ones determined for the precision testing and comprise of %CV ≤35.0% for the titration assay and the 3-fold limitation between the titration data as well as for 80% of the tested reagents in the TZM-bl assay.

**Table 1:** Parallel performed titration assays to demonstrate the stability of the pseudovirus stock at RT and to determine the 3-fold acceptance limit between directly frozen viruses and the ones incubated at RT.

Pseudovirus	Dilution at an RLU of 150,000
ZM249M.PL1 incubation at RT for 2 hrs and 20 min	38
ZM249M.PL1 incubation at RT for 2 hrs and 20 min	34
Average incubation at RT for 2 hrs and 20 min	36
ZM249M.PL1 directly frozen at -80°C	25
ZM249M.PL1 directly frozen at -80°C	40
Average directly frozen at -80°C	33
acceptance limit	11 to 99

### Statistical analysis

Two formally validated Excel-based macros were utilized (I) to determine the dilution to achieve 150,000 RLU within the titration assay and (II) to calculate the IC50 values based on the measured luminescence data within the neutralization assay [13]. OriginPro 2016G has been used for the Kolmogorov-Smirnov normality test and the one-way ANOVA according to Bonferroni method of six to eight individual values of the titration data with a selected significance level of *p*>0.05. The mean, standard deviation, %Acc and %CV was calculated with Microsoft Excel 2013.

## Results

The validation of the automated system for aliquoting occurred according to the ICH Q7 guidelines (http://www.ich.org) and EC guide to GMP Annex 15 (http://ec.europa.eu) with the selected key parameters accuracy, precision, specificity and robustness. Table 2 summarizes the validation parameters together with the performed validation experiments and the respective limits determined within the optimization experiments. Central Quality Assurance Unit (CQAU) of CAVD/CAVIMC has overseen the validation of the automated procedure and the system. All related documents such as the optimization report, validation plan authorized by the quality assurance unit, validation report and the related SOPs are created and constitute the documented evidence of the entire validation procedure.

**Table 2:** Experimental design for each validation parameter tested, and the respective acceptance limits for the evaluation of the automated system for HIV pseudovirus aliquoting.

Validation Parameter	Test Description	Acceptance Criteria
Accuracy	Pipettor volumes (12 or 24 gravimetrical measurements, 8 tips)	%Acc ≤5.0%
	Volume measurement of the ultrasound sensors 10 measurements of an aluminum block)	%Acc ≤5.0% Detected volume between 950 μl and 1050 μl
	Volume measurement of the ultrasound sensors (10 measurements of 1 rack automatically aliquoted)	%Acc ≤5.0% Detected volume between 950 μl and 1050 μl
Precision	Pipettor volumes (12 or 24 gravimetrical measurements, 8 tips)	%CV ≤0.75%
	Volume measurement of the ultrasound sensors (10 measurements of an aluminum block)	%CV ≤0.75%
	Volume measurement of the ultrasound sensors (10 measurements of 1 rack automatically aliquoted)	%CV ≤3.0%
	Pipettor volumes (photometric test)	%CV ≤3.0%
	Large-scale automated aliquoting (at least 10 racks) of a low, middle and high titer HIV-1 pseudovirus	Dilution in TCID assay at 150,000 RLU within 3-fold %CV ≤35.0 at the dilution of 150,000 RLU IC50 titers within 3 fold for ≥80% of the test reagents
	Five repeats of the same virus	Dilution in TCID assay at 150,000 RLU within 3-fold %CV ≤35.0 at the dilution of 150,000 RLU
Specificity and Robustness	Stability of the pseudovirus during the period of automated aliquoting	Dilution in TCID assay at 150,000 RLU within 3-fold
	Automatically vs. manually aliquoted virus stock	Dilution in TCID assay at 150,000 RLU within 3-fold %CV ≤35.0 at the dilution of 150,000 RLU IC50 titers within 3 fold for ≥80% of the test reagents
	Sterility test during operation	No microbial growth
	Sterility of the worktable	No microbial growth
	Disinfection efficiency of 0.4% peracetic acid disinfectant solution of the Ismatec tubing system	No microbial growth

### Accuracy

Within the validation study the verification of the accuracy refers to (I) pipetting volume in general and (II) the distributed volume during operation detected with the ultrasound sensors. The accuracy of the pipetting volume of each channel was verified by gravimetrical measurement of the selected volumes 100 μl and 500 μl. The volume of 500 μl is of particular interest because the target volume of 1000 μl during the automated aliquoting process is achieved by two pipetting tasks of 500 μl. Based on the gravimetrical results, which were lower than the selected target volume, the %Acc of the 12 times measurement for 100 μl was -3.4% and for 24 times measurement for the 500 μl -1.5% (S9 and S10 Tables) and therefore in the pre-defined limits for %Acc ≤ 5.0%.

**Fig 2:**
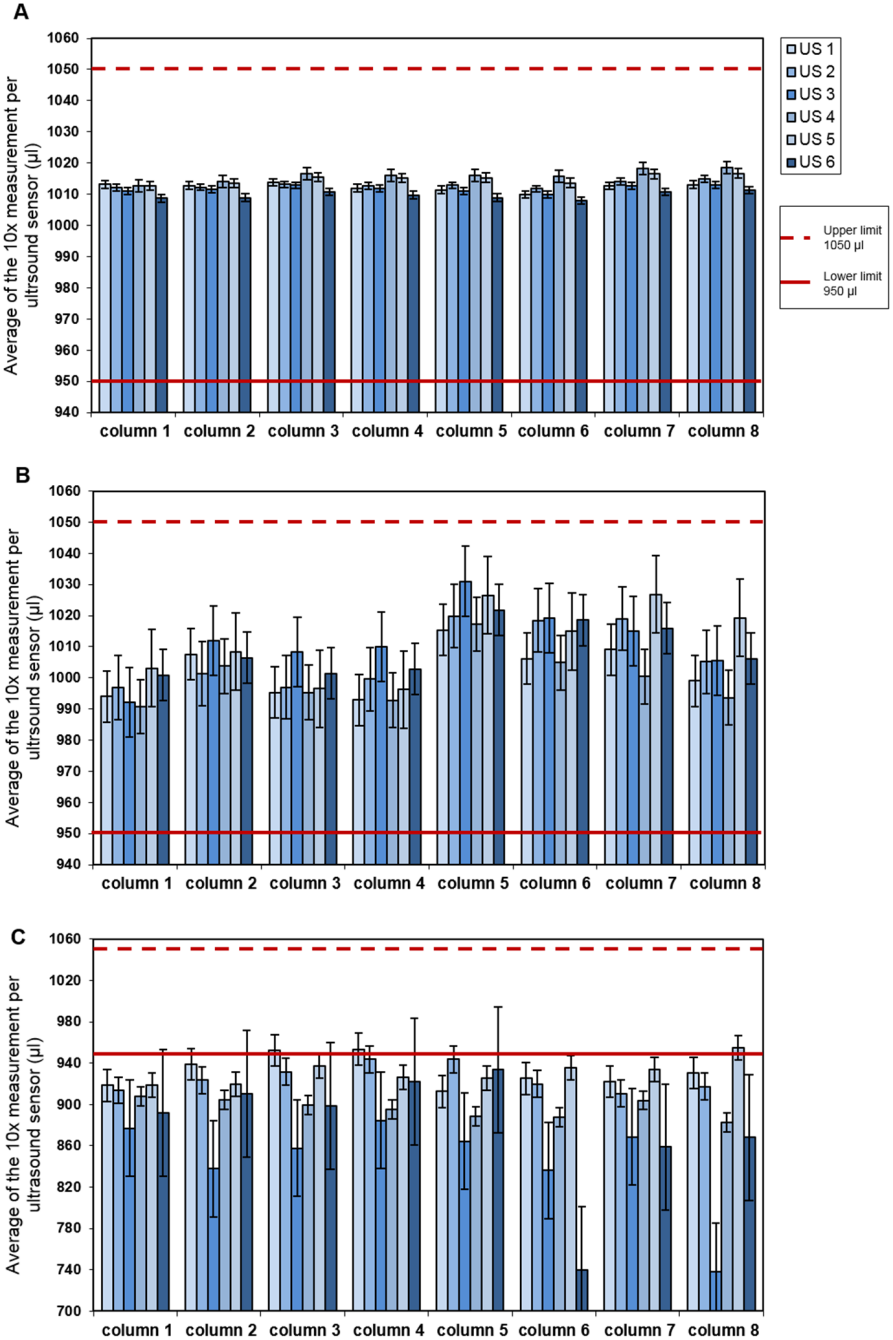
Average volume per column of 10 measurements per ultrasound sensor (US). (A) standardized aluminium block for calibration, (B) one 48-tube rack automatically aliquoted with GM (20% FBS), (C) one 48-tube rack manually aliquoted with GM (20% FBS); the acceptance limit is defined between 950 and 1050 μl.

The accuracy of the ultrasound sensors for the online liquid detection was validated first to basically guarantee the reliability of their measurement within an ongoing process. The calibration occurred with a standardized produced aluminum block in a 10-times measurement (Cellomation) representing the filling level of each position of the cryovial with the target volume of 1000 μl. The average volumes of the 10-times measurement from row one to six detected with each ultrasound sensor ranged between 1009.6 μl and 1016.0 μl and a total %Acc of 1.29% (Table 3). As illustrated in Fig 2A the values for each ultrasound sensor are in the specified range of between 950 μl and 1050 μl. Following this, the accuracy of the ultrasound sensors during operation was verified by a 10-times measurement of one rack containing 48 cryovials automatically aliquoted with 1000 μl of growth medium containing 20% FBS. The results illustrated in Fig 2B and Table 3 demonstrated that the detected values were also all between 950 μl and 1050 μl with the accuracy between -0.02% and 1.16% and met the pre-determined acceptance criteria. Even by repetition of this experiment twice the accuracy (range of -0.70% and 0.54%) agreed with the passing criterion of %Acc ≤5.0% (S11 and S12 Tables). Interestingly, the direct comparison to manually distributed GM containing 20% FBS in 2x 500 μl steps using the Multipette Xtreme (Eppendorf) with the 25 ml Combitips Plus (Eppendorf) and the consecutively 10-times measurement with the ultrasound sensors revealed that the %Acc was with -9.86% out of the 5.0% acceptance range defined for the automation. The average of the detected volume was predominantly lower than 950 μl (Table 3). Only three average values of the 10-times measurement reached the 950 μl limit: US1 in column three with 952 μl, US1 column four with 954 μl and US5 column eight with 955 μl (Fig 2C). The repetition of this measurement as an independent task showed a similar outcome with a total inaccuracy of -9.16% (S13 Table). However, the manual method was not adapted for the volume detection with the ultrasound sensors and differed from the automated proceeding. While for the standard manual technique the aliquoting was performed consecutively by touching the sidewall of the cryovial, within the automated process the liquid was distributed with eight channels in parallel in a free-dispense manner utilizing optimized settings, i. a. of the liquid classes for each individual channel for an accurate distribution of the respective liquid, consisting of the virus stock containing GM with 20% FBS in total or the attaching of the disposable tips with a consistent direction of motion and force. Nevertheless, the manual procedure was still selected as control within this study, because this is the standard proceeding for the virus stock aliquotation. Furthermore, this demonstrates the difficulty to define an adequate reference control within the validation process.

**Table 3:** Average volume (μl), standard deviation (SD), precision (%CV) and accuracy (%Acc) of the 10-times measurement with the ultrasound sensors of the standardized aluminium block, one 48-tube rack automatically and one 48-tube rack manually aliquoted with GM plus 20% FBS.

	Standardized aluminum block							
	US1	US2	US3	US4	US5	US6	%CV in total	%Acc in total
Average (μl)	1012.3	1013.0	1011.7	1016.0	1014.9	1009.6	-	-
SD	1.24	1.04	1.06	1.94	1.45	1.20	-	-
%CV	0.12	0.10	0.11	0.19	0.15	0.12	-	-
%Acc	1.23	1.30	1.17	1.60	1.49	0.96	-	-
-	-	-	-	-	-	-	0.45	1.29
	GM + 20% FBS automatically aliquoted							
	US1	US2	US3	US4	US5	US6	%CV in total	%Acc in total
Average (μl)	1002.4	1007.1	1011.6	999.8	1011.5	1009.3	-	-
SD	8,23	10,21	11,16	8,76	12,39	8,27	-	-
%CV	0.82	1.02	1.12	0.88	1.24	0.83	-	-
%Acc	0.24	0.71	1.16	-0.02	1.15	0.93	-	-
-	-	-	-	-	-	-	1.12	0.70
	GM + 20% FBS manually aliquoted							
	US1	US2	US3	US4	US5	US6	%CV in total	%Acc in total
Average (μl)	931.7	925.5	845.6	896.3	931.4	877.9	-	-
SD	15.30	12.92	46.61	9.16	11.74	61.12	-	-
%CV	1.53	1.29	4.66	0.92	1.17	6.11	-	-
%Acc	-6.83	-7.75	-15.44	-10.37	-6.86	-12.21	-	-
-	-	-	-	-	-	-	5.62	-9.86

### Precision

#### Precision of the volume

The precise pipetting task of the hardware components of the LiHa, including the connected peristaltic pump and syringes, was analyzed based on the data from the multiple gravimetrical measurement with the eight DiTis (Tecan). The calculated values for the %CV range between 0.48% for the 500 μl and 0.47% for the 100 μl met the pass criterion of less than 0.75% (S9 and S10 Tables). Furthermore, with regard to a monthly quality test of the precision of the distributed volume, a photometric test had been implemented by distributing 500 μl distilled water (LONZA, Bulk Packed Sterile WFI, density: 1.0 g/ml) plus 500 μl of Orange G (Applichem, stock solution 0.1 g/ l) with each channel of the automated system in triplicate followed by the photometric measurement. The precision of the automated distributed pipetting volume was confirmed by the determined %CV between 1.1% and 1.2% (Tables A, B and C in S14 Table) and consequently within the pre-defined acceptance criterion of ≤3.0%.

In order to verify the precision of the ultrasound sensors for the liquid detection inside each individual cryovial the data from the calibration measurement using the standardized aluminium block (Cellomation) have been analyzed, given the results for the %CV between 0.10% and 0.19% and therefore within the defined acceptance limit of %CV ≤0.75% (Table 3). After that, one rack containing 48 cryovials was automatically aliquoted with 1000 μl of growth medium containing 20% FBS in total and measured 10-times consecutively. As indicated in Table 3 the %CV for the detected values per ultrasound sensor were between 0.82% and 1.24%, also meeting the passing criterion of %CV ≤3.0%. Even after repetition of this control measurement twice, the variability between the measurements of the ultrasound sensors was low and agreed with a determined intermediate precision of 0.76% well within the pre-defined limit of %CV ≤3.0% (S15 Table). As indicated for the precision the results of the 10-times measurement of the manually distributed GM containing 20% FBS revealed also a generally higher variability of the volume detected by the ultrasound sensors and was for US3 with 4.66% and US6 with 6.11% (Table 3) out of the %CV ≤3.0% range determined for the automation. As mentioned above this is due to the not completely identical manual and automated aliquoting protocol and suggests to the difficulty that the manual one cannot be considered as an adequate standard in this case.

#### Large-scale automated aliquoting of low, middle and high titer viruses

**Fig 3:**
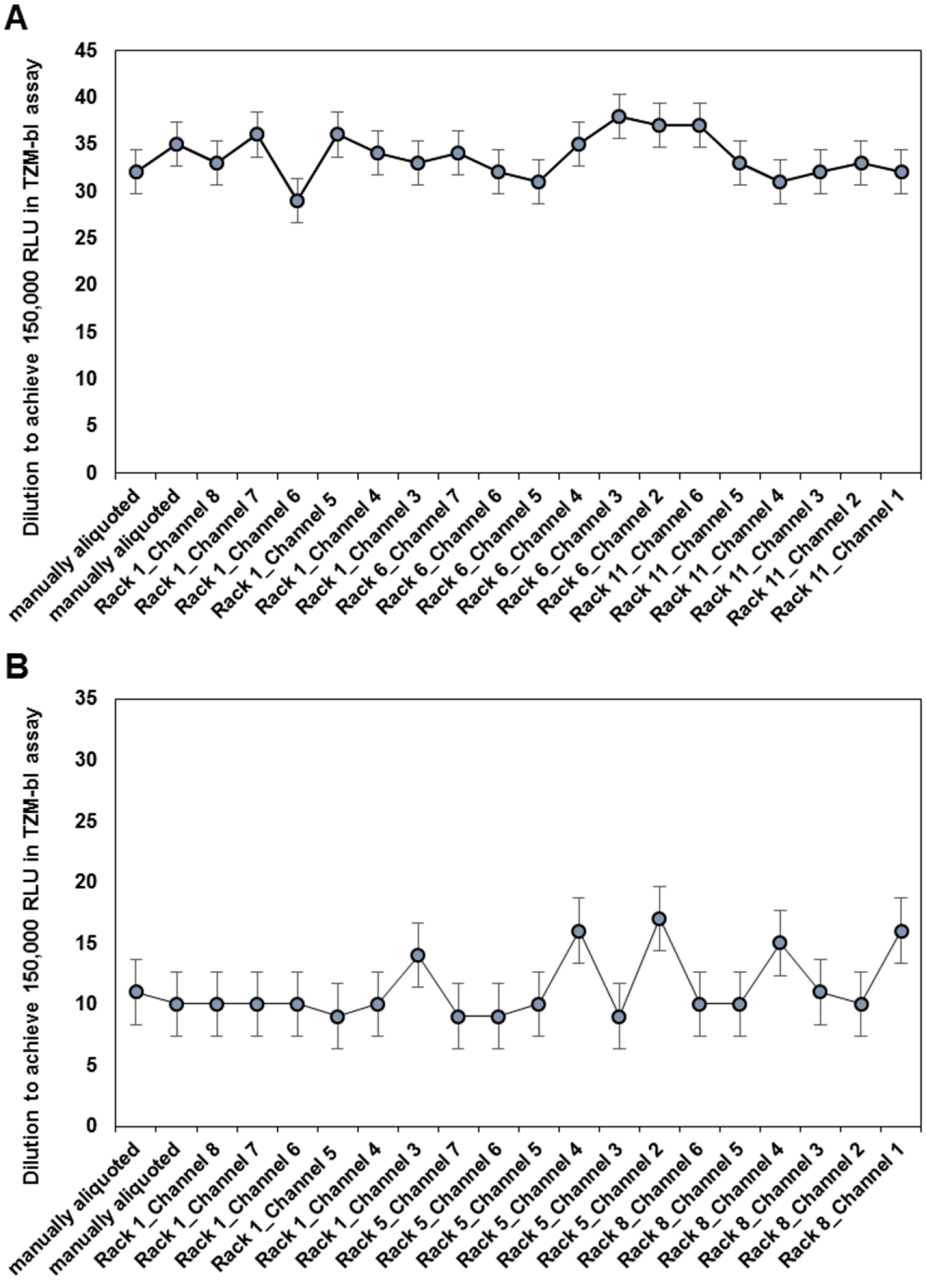
Titration data of the pseudovirus RHPA4259.7 displaying each channel from the first, middle and last rack aliquoted automatically. (A) first harvest with the defined 3-fold range between 11 and 102 and the %CV of 7.0, (B) second harvest with the defined 3-fold range between 4 and 33 and the %CV of 23.5.

The established process for automated aliquoting was validated by large-scale automated aliquotation experiments (at least ten racks) of one low titer (SF162.LS), one middle titer (RHPA4259.7) and one high titer (QH0692.42) pseudovirus stock. The results of the titration assays of one vial of the first, the middle and the last distributed rack indicated that the pseudovirus dilutions at 150,000 RLU are for all three pseudoviruses in 3-fold range compared to the pseudovirus stocks before the automated aliquoting process, which have been aliquoted manually (Table 4). In addition, titration experiments, displaying each channel of the automated system from the first, the middle and the last distributed rack, were performed for the first and the second harvest of the pseudovirus RHPA4259.7. In Fig 3 the titration data showed slight variances at the dilution of 150,000 RLU, which were equally distributed, thus a bias of one of the channels could be excluded and were with 7.0% for first harvest and 23.5% for the second within the acceptable limit of %CV ≤35.0%. Furthermore, the established 3-fold range of the automatically aliquoted virus stock against the manually aliquoted was met for both harvests. Finally, the concordant quality of the virus stocks with different infectivity aliquoted with the automated system was verified successfully in parallel TZM-bl assays by their fitting into the 3-fold range of the neutralization titers in comparison to the manually aliquoted reference viruses (Table 5).

**Table 4:** Precision of automatically aliquoted HIV-1 pseudovirus stocks in large-scale of low, middle and high titer viruses. Comparison of the dilution at an RLU of 150,000 to the manually aliquoted reference virus with the pre-defined 3-fold range as pass criterion.

Pseudovirus	Dilution to achieve 150,000 RLU in TZM-bl assay
SF162.LS Rack No.1 Position F5	4
SF162.LS Rack No.5 Position D4	5
SF162.LS Rack No.10 Position A6	4
SF162.LS manually aliquoted	5
acceptance limit 3-fold range	2 to 15
RHPA4259.7 Rack No.1 Position F8	33
RHPA4259.7 Rack No.6 Position D5	31
RHPA4259.7 Rack No.11 Position A1	32
RHPA4259.7 manually aliquoted	32
acceptance limit 3-fold range	11 to 96
QH0692.42 Rack No.1 Position F5	90
QH0692.42 Rack No.5 Position B3	120
QH0692.42 Rack No.10 Position A7	90
QH0692.42 manually aliquoted	95
acceptance limit 3-fold range	32 to 285

**Table 5:** Quality control of the precision of large-scale HIV-1 pseudovirus stocks. Neutralization titer comparison of automatically and manually aliquoted viruses by assaying five defined test reagents.

	IC50 values (μg/ml) of virus stocks determined with HIV-1 neutralizing test reagents				
Pseudovirus	sCD4	IgG1b12	2F5	4E10	TriMab
SF162.LS (Rack No. 10 Position A1)	0.16	0.05	3.00	8.76	0.20
SF162.lS (manual reference stock)	0.14	0.04	2.48	6.93	0.09
acceptance limit 3-fold range	0.05 to 0.42	0.01 to 0.12	0.83 to 7.4	2.31 to 20.79	0.03 to 0.27
RHPA4259.7 (Rack No. 11 Position A3)	3.10	0.13	23.58	16.83	0.34
RHPA4259.7 (manual reference stock)	3.57	0.14	21.16	17.83	0.35
acceptance limit 3-fold range	1.19 to 10.71	0.05 to 0.42	7.05 to 63.48	5.94 to 53.49	0.12 to 1.05
QH0692.42 (Rack No. 10 Position A1)	2.31	0.85	3.58	6.90	1.18
QH0692.42 (manual reference stock)	2.35	0.53	1.85	4.98	0.92
acceptance limit 3-fold range	0.78 to 7.05	0.18 to 1.59	0.62 to 5.55	1.66 to 14.94	0.31 to 2.76

#### Inter-assay precision

With the aim to validate the precision of the automated aliquoting procedure, one rack of the pseudovirus stock X1632_S2_B10 was aliquoted in five repeats consecutively on the automated system and compared to the respective manually distributed one, aliquoted at the same time. There was no difference whether the virus stock was distributed automatically or manually in five independent experiments. Only a slight reduction was observed for the dilutions at 150,000 RLU from set up one and two compared to repeat numbers three to five with an inter-assay variability of 7.8% (%CV), due to the assay performance on different time points using a different TZM-bl stock. Nevertheless, for all automated aliquoting tasks of the pseudovirus X1632_S2_B10 the dilutions at 150,000 were in 3-fold range with an intra-assay variance ranging from %CV 1.7–3.7%, which agree well with the acceptance limit of %CV ≤35.0% (Table 6). Therefore, the precision of the automated pseudovirus aliquotation could be validated successfully.

**Table 6:** Repeatability precision. Mean of the titration data in duplicate (dilution at 150,000 RLU), SD and %CV of five repeats of the pseudovirus X1632_S2_B10 aliquoted successively with the automated system against the manually filled reference stock and the 3-fold range as acceptance criterion.

Repeat No.	Mean manual	Mean automated	Acceptance limit 3-fold range	SD	%CV
1	40	42	13 to 120	1.5	3.7
2	43	41	14 to 129	1.0	2.5
3	48	47	16 to 144	1.2	2.4
4	48	48	16 to 144	0.8	1.7
5	48	47	16 to 144	0.8	1.7

### Robustness

#### Robustness of the virus stocks distributed with the automated system

The duration for aliquoting of a large-scale virus stock with the automated system takes 1 h and 50 min for eleven racks containing 48 cryovials. To demonstrate the stability of the virus stock during the entire aliquoting period the pseudovirus RHPA4259.7 was produced in large-scale (40 T-75 flasks) and aliquoted in eleven racks with the automated system. As control, 10 samples were aliquoted manually and directly frozen at -80°C, and 20 aliquots were filled manually before the automated aliquoting procedure and frozen together with the racks from the automation task. Despite a modest decrease at the dilution of 150,000 RLU up to 1.3-fold compared to the immediately frozen samples (Table 7), the stability during the complete period of the automated aliquoting process was approved with a consistent quality of pseudovirus stock which was demonstrated in the neutralization assay of one sample of the last rack (Table 5).

**Table 7:** Titration data of the pseudovirus RHPA4259.7. Automatically aliquoted pseudovirus and the manually filled virus before the automated aliquoting procedure compared to manually filled virus directly frozen with the allowed 3-fold acceptance limit.

Pseudovirus	Dilution at an RLU of 150,000
RHPA4259.7 Rack No.1 Position F7	30
RHPA4259.7 Rack No.11 Position A2	30
RHPA4259.7 before automated aliquoting	35
RHPA4259.7 directly frozen at -80°C	40
acceptance limit 3-fold range	13 to 120

#### Direct comparison of an automatically and manually distributed virus stock

**Fig 4:**
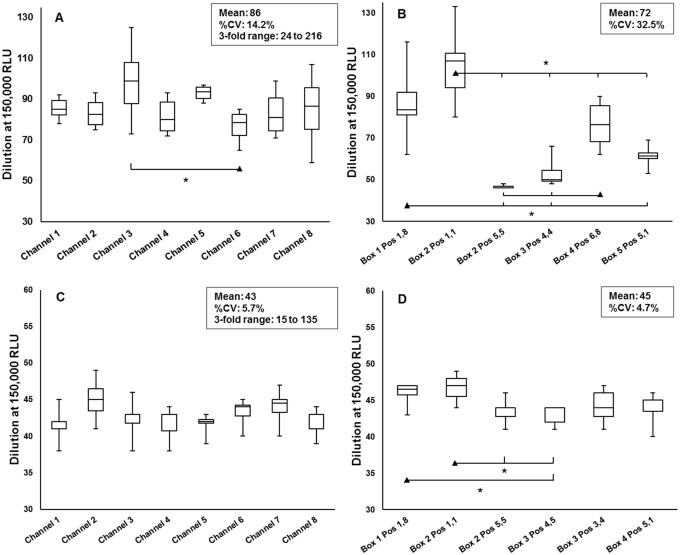
Variability of the titration data at the dilution of 150,000 RLU. (A) PVO.4 first harvest automatically aliquoted, (B) PVO.4 first harvest manually aliquoted, (C) PVO.4 second harvest automatically aliquoted, (D) PVO.4 second harvest manually aliquoted. *Statistical difference ▲in comparison to **|** with *p*<0.05.

The direct comparison of the automated and manual aliquotation method was assessed with the first and the second harvest of the large-scale produced pseudovirus PVO.4 (40 T-75 flasks), each half aliquoted automatically or manually. By testing one cryovial from each automatically aliquoted rack displaying each channel and one cryovial frozen in various cryoboxes at different positions inside the box (outer corner, outer and inner) of the manual filled samples, concordance with the 3-fold boundary was achieved by matching the general variation limit of %CV ≤35.0% (Fig 4). By passing the parallel performed TZM-bl assay test of the last automatically aliquoted cryovial against the historical manually filled one confirmed again the quality of the automated aliquoting procedure (S16 Table). Therefore, as overall outcome of this validation study, the uniformity of the automated aliquoting procedure against the manual one was verified successfully.

**Fig 5:**
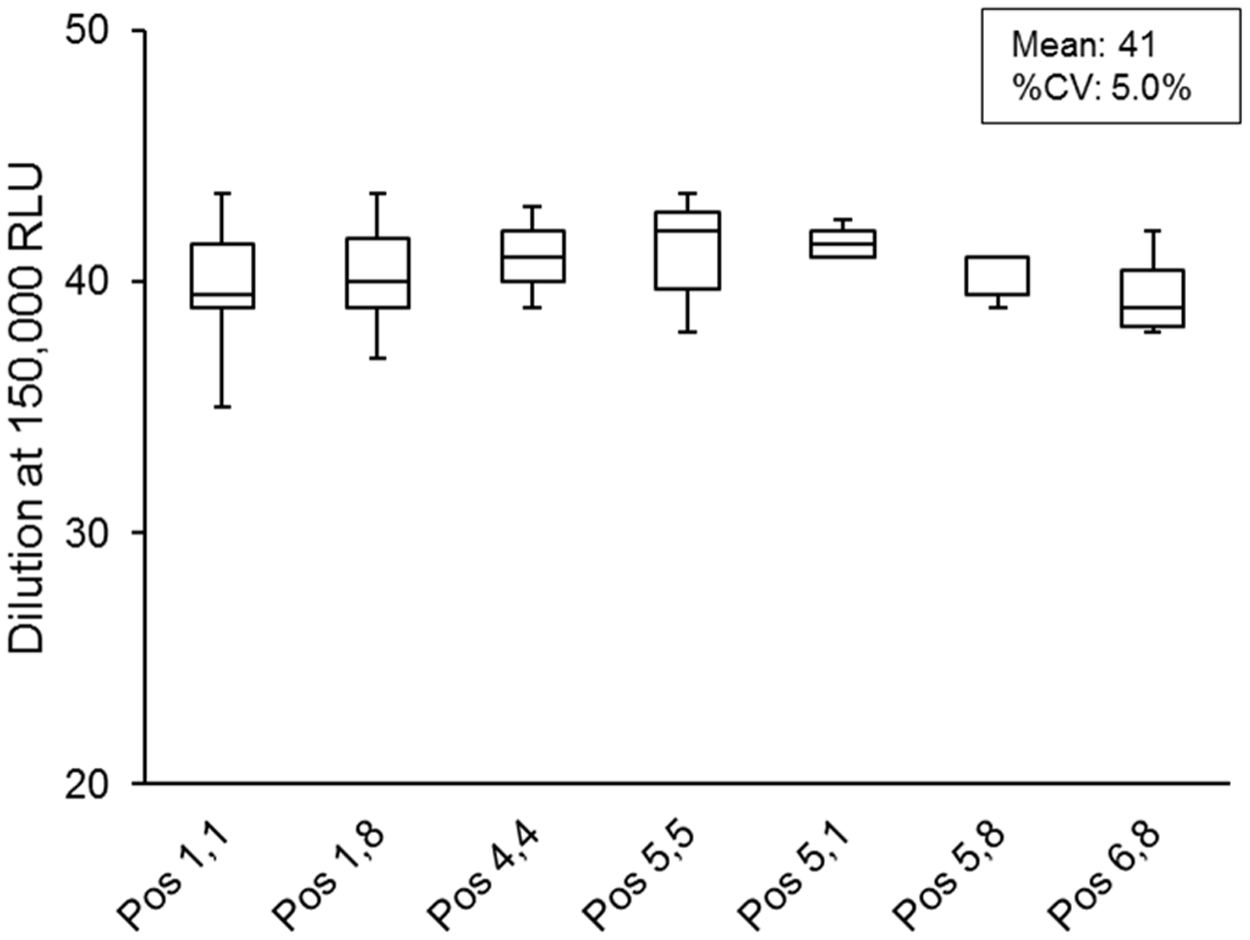
Variability of the titration data at the dilution of 150,000 RLU for the pseudovirus SS1196.1 frozen according to the automated procedure in a plastic rack at -80°C.

Further, the variation among each channel of the automatically aliquoted samples and difference of the manually distributed one were characterized by one-way ANOVA. It was found, that for the majority of the tested positions no significant differences could be determined. Among each channel of the automated distribution only for the first harvest between channel six (mean dilution at 150,000 RLU: 1:77) and channel three (mean dilution at 150,000 RLU: 1:99) a significant difference could be detected, demonstrating that the quality of the pseudovirus stocks is not influenced by the automated aliquoting procedure (Fig 4A and 4C). The individual analysis of the cryovials following the manual filling procedure frozen at different positions inside the cryoboxes indicated some significant differences, especially between the cryovials at the outer edge position (Pos 1,1 and Pos 1,8) and the inner position (Pos, 4,4, Pos 4,5 and 5,5) within the cryobox (Fig 4B and 4D). Further, a significant difference of the first harvest of the outer position 6,8 was significantly higher than the samples from the inner position (Pos 5,5 and Pos 4,4). These differences might be due to the utilization of different freezing procedures of the automatically and manually aliquoted viruses. The automated ones were frozen in the 48-way plastic racks in one compartment of the -80°C freezer and the manual one more tightly packed in carton cryoboxes closed with a cover and frozen in a steel rack within the -80°C freezer. To overcome this effect of different freezing methods an additional titration assays was performed. The pseudovirus stock SS1196.1 was manually aliquoted, frozen at -80°C overnight in one plastic rack with one cm distance between each cryovial, transferred to the carton cryoboxes and then stored at the steel racks. The one-way-ANOVA of the titration data clearly demonstrate no significant differences between the samples, independent from the position during freezing and the storage position within the carton cryobox (Fig 5).

#### Sterility

Sterile and standardized operation conditions are mandatory for the automated aliquoting process. The Laminar Flow Hood Type WK 12.19-S3 (BDK) guarantees the protective surrounding for the pseudoviruses as well as for the environment and personnel. During the validation process the sterility of the worktable was verified using Tryptic Soy Agar plates (Heipha), which were incubated without lid on and under the worktable and at the position where the virus supply bottle is located. Further, the system liquid was also tested with the Tryptic Soy Agar plates. As a result, in none of these plates an indication of microbial growth could be detected (Tables A and B in S17 Table). Another crucial component in the aliquoting procedure, which needs to be sterile, is the tubing system for virus transfer from the virus supply bottle to the trough on the worktable integrated into the Ismatec tubing pump. Although the tubings, consisting of Pharmed^®^ tubes (Saint-Gobain Performance) and the connected Ismatec 3-Stop-Tubing (Tygon^®^ Silikon 3350 Platin) are designed as disposables to exclude an accidental cross-contamination between different virus stocks, they must be autoclaved, before their insertion into the cassette of the pump. Following a short cleaning procedure with 70% Ethanol, sterile water and a flushing step at the end to empty the tubes, the sterility was verified with a modified sterility test by pumping sterile medium through these tubes into the trough on the worktable and the automated aliquotation of one rack (1 ml/ per cryovial). The microscopic evaluation of 15 ml of this medium after transfer into a T-75 flask and the incubation at 37°C for 4 days showed no incidence of a microbial contamination (Table C in S17 Table), which successfully confirmed the transfer of liquids through the tubing system under sterile conditions during operation.

## Discussion

In this article, the key experiments necessary for the validation of the automated system for aliquoting of HIV-1 pseudoviruses according to GCLP have been outlined to test the following validation parameter: accuracy, precision, specificity and robustness. This allows the standardized and operator independent distribution of virus stocks from small-scale to bulk amounts of HIV-1 pseudovirus stocks between 48 ml and 960 ml with a precise and reproducible outcome, given the advantage of automation to facilitate labor-intensive and repetitive work [19]. In the first place, the equivalence of the automated aliquoting procedure in comparison to the manual one was demonstrated successfully. There was no difference in the titration data and the neutralization profiles, nevertheless, if the virus stocks were aliquoted with the automated system or manually (Fig 4 and Tables 3–6). Thereby, the automated proceeding gives here the important advantage to reduce the aliquoting time up to 10 to 20% compared to the manual method which is related to an ameliorated handling of the samples at room temperature. One additional beneficial aspect of the automated procedure, particularly with regard to the integration into a quality assurance program, is the online monitoring of the complete aliquoting process in real-time and the related traceability of each individual vial within the respective aliquoting task. This is realized by the integration of six ultrasound sensors for measuring the liquid level per cryovial as well as the cap status following the automated re-capping procedure. However, this makes the establishment of suitable quality controls even more important and have been implemented within the validation period. To control for the liquid level detection of the ultrasound sensors, a 10-times measurement of one rack automatically aliquoted with medium plus 20% FBS is performed monthly, and once a year the maintenance including the 10-times measurement of the aluminum block is also performed. In addition, the precision of the pipetting volume is controlled regularly, once a month with a photometric test and twice a year with a gravimetrical measurement. For the performed test until now, the acceptance limits outlined in Table 2 were always met, indicating that the system is valid for the precise and reliable aliquotation of HIV-1 pseudovirus stocks, and a correct volume detection with the ultrasound sensors.

The hallmark of the automated aliquoting system is the lower operator dependency, which is a major issue for manual proceedings. This is mediated by the fact, that the automated system works in a closed environment, applying for the liquid handling pre-defined settings with specified volumes and action points, e.g. for the attachment of DiTis on each channel. Especially with respect to the aliquoting procedure by itself the process parallelization for the liquid handling with eight fixed channels reduce the variability of the distributed volume per cryovial in comparison to the manual method, where the cryovials are filled consecutively. The operator dependencies play also an important role when cell-based assays are applied during the validation process, like in this validation study the titration assays using the TZM-bl cells. Several factors influence the assay results on the biological site, such as the cell cycle or the passage number of the cells, which influence the related number of CD4 and the co-receptors CCR5 and CXCR4 on the cell surface [13]. Besides the biological aspects operator specific variances during assays performance are also playing a crucial role as inter-operator variability between different operators as well as the intra-assay variability, if one person is repeating the experiment for several times and were also observed in the validation phase, e. g. for the virus RHPA4259.7 the % CV with respect to the titration experiments laid between 7.0% and 23.3% and for the direct comparison between the manual and automated procedure 14.2% and 32.5%. These variances in cell-based assays are a common phenomenon and were also described in detail during the validation of the TZM-bl assay with an established acceptance limit %CV ≤45% and an error rate of 20% for the 50% inhibitory dose (ID50) values [13]. To overcome the observed variability obstacles within the titration experiments, there is a strong need to transfer this cell-based assay to an automated system to exclude any operator dependencies and make the outcome more reliable. The feasibility to automate a TZM-bl-based protocol was initially described by Sarzotti-Kelsoe et al. in 2014 [13] with the implementation and validation of the TZM-bl assay with increased throughput, by the adaptation of the assay to an automated 384-well format. However, the detected variability of the titration assay outcomes have no impact on the basic finding of this validation study that the automated platform for aliquoting HIV pseudoviruses works in accordance to the manual procedure, with an additional confirmed quality of the virus stocks in neutralization assays.

Furthermore, the data of one-way ANOVA analysis regarding the direct comparison of the automated and manual distributed virus stocks revealed that the freezing conditions for the automation is more advantageous compared to the manually processed samples (Fig 4). While during the manual freezing bulk amounts of virus stock samples were brought into a smaller area at the same time and packed tightly in carton boxes, within the automation method the samples were frozen in a 48-tube rack in one complete compartment of the -80°C freezer. These variances during freezing procedures are accompanied with differences in the cooling rate and reflected in the outcome of the titration analysis. But also within one carton box different temperatures during freezing process must exist, which is displayed by a significant higher titer of the samples at outer position compared to the inner one (Fig 4B and 4D). By restoring these variabilities in titer, we adapted the freezing procedure of the manually aliquoted to the automated one (Fig 5), confirming the primary assumption that the cooling rate plays a crucial role on the stability of the infectious pseudovirus particles, a known feature for the quality controlled cryopreservation of cells [20]. These findings are very interesting and need to be investigated in detail to further improve the quality of the HIV-1 virus stocks.

## Conclusion

In conclusion, the occurrence of variabilities in the assay output clearly demonstrate the importance of the standardized preparation and handling of HIV-1 pseudoviruses as valuable test reagent and strongly emphasize the implementation of automation within the laboratory field to reduce operator and environmental influences to a minimum. To close the circle of the automated production procedure next to the automated platform for HIV-1 pseudovirus preparation [7] the automated aliquoting system has been established and has successfully been validated in accordance to GCLP. Thus, this novel approach of standardized, automated production and aliquoting will support upcoming pre-clinical and clinical studies where large-scale amounts of pseudoviruses are needed.

## Supporting information

S1 Table Results of the gravimetrical measurement for the selected volume of 100 μl plus the average (μl), standard deviation (SD), precision (%CV) and accuracy (%Acc). (PDF)

S2 Table Results of the gravimetrical measurement for the selected volume of 500 μl plus the average (μl), standard deviation (SD), precision (%CV) and accuracy (%Acc). (PDF)

S3 Table Individual values of the 10-times measurement with the ultrasound sensors (US) of the standardized aluminium block plus the average (μl), standard deviation (SD), precision (%CV) and accuracy (%Acc). (PDF)

S4 Table Individual values of the 10-times measurement with the ultrasound sensors (US) of one 48-tube rack automatically aliquoted with GM containing 20% FBS plus the average (μl), standard deviation (SD), precision (%CV) and accuracy (%Acc). (PDF)

S5 Table Titration data of the pseudovirus (A) ZM214M.PL15 and (B) SF162.LS of automatically aliquoted pseudovirus and the manually filled virus before the automated aliquoting procedure to set the 3-fold acceptance limit and the intra-assay variability (%CV) ≤35.0%. (PDF)

S6 Table Titration data of the pseudovirus SF162.LS of (A) Batch #1 and (B) Batch #2 to set the acceptance limit for the intra-assay variability (%CV) ≤35.0%. (PDF)

S7 Table Parallel performed neutralization assays to determine the acceptance limit to verify the integrity/quality of the automatically aliquoted HIV-1 pseudovirus stocks. Compared are the neutralization titers of the automatically and the manually aliquoted reference viruses by assaying five defined test reagents.
(PDF)

S8 Table Titration data of the pseudovirus (A) CH110.2 and (B) Q842.d12 incubated under different conditions before storage at -80°C. (PDF)

S9 Table Individual results of the validation experiment of the gravimetrical measurement for the selected volume of 100 μl plus the average (μl), standard deviation (SD), precision (%CV) and accuracy (%Acc). (PDF)

S10 Table Individual results of the validation experiment of the gravimetrical measurement for the selected volume of 500 μl plus the average (μl), standard deviation (SD), precision (%CV) and accuracy (%Acc). (PDF)

S11 Table Individual values of the 10-times measurement with the ultrasound sensors (US) of one 48-tube rack automatically aliquoted with GM containing 20% FBS plus the average (μl), standard deviation (SD), precision (%CV) and accuracy (%Acc). (PDF)

S12 Table Individual values of the 10-times measurement with the ultrasound sensors (US) of one 48-tube rack automatically aliquoted with GM containing 20% FBS plus the average (μl), standard deviation (SD), precision (%CV) and accuracy (%Acc). (PDF)

S13 Table Individual values of the 10-times measurement with the ultrasound sensors (US) of one 48-tube rack manually aliquoted with GM containing 20% FBS plus the average (μl), standard deviation (SD), precision (%CV) and accuracy (%Acc). (PDF)

S14 Table Average OD, standard deviation (SD) and precision (%CV) of the photometric test. Shown are the results of three 48-well plates (A, B and C), whereby each channel distributed 6 times 500 μl distilled water and 500 μl Orange G.
(PDF)

S15 Table Intermediate precision after 10-times measurement with the ultrasound sensors (US) of one rack automatically aliquoted. (PDF)

S16 Table Parallel performed neutralization assays to verify the integrity of the large-scale prepared virus stock PVO.4 after the automated aliquoting process. Compared are the neutralization titers of the automatically and the manually aliquoted historical reference viruses by assaying five defined test reagents with the defined 3-fold acceptance limit.
(PDF)

S17 Table Summary of the visual and microscopic evaluation of the sterility tests for (A) the worktable, (B) the system liquid and (C) the virus supply tubing system. (PDF)
