# Supplementary material for: Validation of an automated system for aliquoting of HIV-1 Env-pseudotyped virus stocks
Source: PLoS One. 2018 Jan 4;13(1):e0190669. doi: 10.1371/journal.pone.0190669 (PMC5754138; doi:10.1371/journal.pone.0190669)
Supplement: S1 Table — (PDF) [file pone.0190669.s001.pdf]

S1 Table. Results of the gravimetrical measurement for the selected volume of 100 µl plus the Average (µl), Standard Deviation (SD), Precision (%CV) and Accuracy (%Acc).

	Channel 1	Channel 2	Channel 3	Channel 4	Channel 5	Channel 6	Channel 7	Channel 8	Average	SD	%CV
1	99,528	98,902	99,508	99,132	99,310	99,470	99,374	99,280	99,3	0,2	0,2
2	98,928	98,344	98,924	98,504	98,656	99,018	98,750	98,904	98,8	0,2	0,2
3	98,670	97,870	98,516	98,602	98,404	98,596	98,278	98,562	98,4	0,3	0,3
4	98,496	97,720	98,412	97,892	98,152	98,328	98,220	98,330	98,2	0,3	0,3
5	98,148	97,490	98,132	97,830	97,566	98,362	97,924	98,406	98,0	0,3	0,3
6	98,090	97,474	98,078	97,636	97,594	98,022	97,804	97,966	97,8	0,2	0,2
7	97,964	97,180	97,878	97,550	97,472	97,714	97,672	97,828	97,7	0,3	0,3
8	97,894	97,108	98,144	97,418	97,292	97,622	97,828	97,886	97,6	0,4	0,4
9	97,682	97,036	97,764	97,404	97,338	97,534	97,802	97,832	97,5	0,3	0,3
10	97,717	97,052	97,560	97,292	97,046	97,688	97,708	97,726	97,5	0,3	0,3
11	97,608	97,124	97,424	97,172	97,086	97,762	97,506	97,868	97,4	0,3	0,3
12	97,640	96,990	97,788	97,278	97,130	97,586	97,072	97,298	97,3	0,3	0,3
Average	98,2	97,5	98,2	97,8	97,8	98,1	98,0	98,2		**Overall**	
SD	0,6	0,6	0,6	0,6	0,7	0,6	0,6	0,6	**Average**	**SD**	**%CV**
%CV	0,6	0,6	0,6	0,6	0,7	0,6	0,6	0,6	**98,0**	**0,64**	**0,65**

**%Acc**	**-2,0**
**Min**	**97,0**
**Max**	**99,5**
