# Supplementary material for: Validation of an automated system for aliquoting of HIV-1 Env-pseudotyped virus stocks
Source: PLoS One. 2018 Jan 4;13(1):e0190669. doi: 10.1371/journal.pone.0190669 (PMC5754138; doi:10.1371/journal.pone.0190669)
Supplement: S2 Table — (PDF) [file pone.0190669.s002.pdf]

S2 Table. Results of the gravimetrical measurement for the selected volume of 500 µl plus the Average (µl), Standard Deviation (SD), Precision (%CV) and Accuracy (%Acc).

	Channel 1	Channel 2	Channel 3	Channel 4	Channel 5	Channel 6	Channel 7	Channel 8	Average	SD	%CV
1	491,5	491,5	491,5	491,5	491,5	491,5	491,5	491,5	491,5	0,0	0,0
2	491,6	491,6	491,6	491,6	491,6	491,6	491,6	491,6	491,6	0,0	0,0
3	491,6	491,6	491,6	491,6	491,6	491,6	491,6	491,6	491,6	0,0	0,0
4	492,8	492,4	491,5	495,7	491,1	494,8	493,1	494,9	493,3	1,7	0,3
5	492,0	492,3	489,6	492,7	492,0	491,8	489,9	494,3	491,8	1,5	0,3
6	493,3	492,7	493,3	495,2	492,8	494,0	495,7	494,4	493,9	1,1	0,2
7	491,7	491,4	490,0	492,2	491,3	491,2	490,1	493,3	491,4	1,1	0,2
8	491,6	491,8	490,4	492,3	491,4	491,1	490,3	493,4	491,5	1,0	0,2
9	490,2	490,7	497,5	495,0	493,4	498,9	490,9	499,4	494,5	3,8	0,8
10	491,3	491,3	492,7	491,9	492,2	490,7	490,0	492,8	491,6	1,0	0,2
11	494,6	494,3	488,9	492,1	490,8	493,3	492,9	495,2	492,8	2,1	0,4
12	490,2	490,9	500,6	502,3	487,9	493,8	499,2	501,8	495,8	5,8	1,2
13	497,7	497,0	490,7	497,4	488,7	490,8	488,6	492,3	492,9	3,9	0,8
14	491,7	491,4	495,0	496,3	490,4	490,7	490,0	496,1	492,7	2,6	0,5
15	490,6	491,6	491,4	494,6	488,8	492,0	495,4	494,7	492,4	2,3	0,5
16	491,0	490,7	490,2	491,2	488,3	490,0	489,7	492,1	490,4	1,1	0,2
17	491,0	491,0	490,2	491,6	490,0	490,3	489,4	492,3	490,7	0,9	0,2
18	489,0	491,1	495,2	496,4	491,4	493,9	490,6	494,6	492,8	2,6	0,5
19	492,1	492,4	491,8	492,9	491,7	491,6	490,7	493,0	492,0	0,8	0,2
20	491,2	492,1	491,6	492,4	491,7	491,5	490,4	492,9	491,7	0,8	0,2
21	490,5	489,6	489,5	494,2	491,5	493,3	495,8	494,4	492,4	2,4	0,5
22	492,1	492,4	491,8	492,9	492,0	491,7	490,5	493,3	492,1	0,8	0,2
23	491,4	489,9	492,8	496,1	490,9	493,1	493,4	496,0	493,0	2,2	0,5
24	491,2	491,6	491,0	492,1	490,5	490,8	490,0	492,5	491,2	0,8	0,2
Average	491,7	491,8	492,1	493,8	491,0	492,3	491,7	494,1		**Overall**	
SD	1,7	1,5	2,7	2,6	1,4	1,9	2,6	2,4	**Average**	**SD**	**%CV**
%CV	0,3	0,3	0,5	0,5	0,3	0,4	0,5	0,5	**492,3**	**2,34**	**0,48**

**%Acc**	**-1,5**
**Min**	**487,9**
**Max**	**502,3**
