# Supplementary material for: Validation of an automated system for aliquoting of HIV-1 Env-pseudotyped virus stocks
Source: PLoS One. 2018 Jan 4;13(1):e0190669. doi: 10.1371/journal.pone.0190669 (PMC5754138; doi:10.1371/journal.pone.0190669)
Supplement: S3 Table — (PDF) [file pone.0190669.s003.pdf]

S3 Table. Individual values of the 10-times measurement with the ultrasound sensors (US) of the standardized aluminium block plus the Average (µl), Standard Deviation (SD), Precision (%CV) and Accuracy (%Acc).

US-Sensor	Cryovial-Position	1	2	3	4	5	6	7	8	9	10	Average	SD	%CV	%Acc
6	1	1005,27	1004,74	1004,72	1003,80	1002,89	998,72	999,77	998,44	997,31	996,37	1001,20	3,42	0,34	0,12
5	2	1006,21	1002,66	1003,40	1001,58	1000,99	1000,71	998,88	999,19	999,32	999,96	1001,29	2,28	0,23	0,13
4	3	1004,54	1006,26	1006,12	1002,90	1000,81	1000,31	1000,10	997,81	997,07	996,72	1001,26	3,57	0,36	0,13
3	4	1007,47	1006,06	1007,77	1002,72	1002,11	1000,70	1001,19	999,14	999,14	999,28	1002,56	3,39	0,34	0,26
2	5	1004,23	1003,22	1001,37	1000,96	999,62	999,32	997,12	997,09	995,43	995,27	999,36	3,12	0,31	-0,06
1	6	1008,76	1006,36	1005,76	1001,96	1002,03	1002,32	1000,22	999,10	999,18	998,16	1002,39	3,52	0,35	0,24
6	7	1005,94	1006,53	1002,83	1004,27	1002,08	1000,91	1000,28	1000,05	998,53	997,46	1001,89	3,03	0,30	0,19
5	8	1005,40	1003,55	1003,82	1002,91	1002,84	1000,37	999,52	998,94	998,51	999,21	1001,51	2,46	0,25	0,15
4	9	1005,46	1004,28	1004,20	1004,21	1002,29	1000,13	1000,28	1000,22	999,93	996,80	1001,78	2,74	0,27	0,18
3	10	1006,06	1004,28	1003,72	1002,79	1002,58	1002,27	1001,74	1001,11	1000,52	999,86	1002,49	1,85	0,18	0,25
2	11	1003,75	1003,33	1002,75	1002,64	1001,36	998,94	998,57	997,81	996,46	995,37	1000,10	3,04	0,30	0,01
1	12	1007,18	1006,86	1005,32	1002,54	1000,77	1001,32	1001,54	1000,36	999,72	997,73	1002,33	3,14	0,31	0,23
6	13	1007,78	1004,74	1004,16	1004,29	1003,19	1002,80	1002,14	1001,66	1000,48	999,89	1003,11	2,29	0,23	0,31
5	14	1007,20	1005,20	1004,30	1003,84	1002,13	1002,25	1001,65	1001,21	999,90	1002,17	1002,99	2,15	0,21	0,30
4	15	1006,85	1004,54	1003,93	1003,46	1003,55	1003,17	1002,08	1000,98	999,80	1000,18	1002,85	2,15	0,21	0,29
3	16	1007,39	1006,02	1005,06	1005,37	1004,55	1004,12	1003,54	1003,35	1002,10	1001,91	1004,34	1,71	0,17	0,43
2	17	1006,53	1004,44	1003,68	1003,03	1001,86	1000,77	999,14	997,59	996,95	996,63	1001,06	3,42	0,34	0,11
1	18	1009,20	1007,04	1005,19	1003,28	1002,59	1003,28	1001,56	1000,70	1000,59	999,63	1003,31	3,05	0,30	0,33
6	19	1004,55	1004,62	1004,24	1001,85	1000,97	1001,55	1002,74	999,20	999,06	997,27	1001,61	2,53	0,25	0,16
5	20	1006,35	1004,14	1002,48	1002,13	1001,47	1000,84	1000,86	999,09	998,35	999,93	1001,56	2,38	0,24	0,16
4	21	1006,39	1004,67	1004,18	1001,82	1001,83	1002,65	1001,94	1000,61	1000,81	999,85	1002,48	2,04	0,20	0,25
3	22	1007,42	1006,78	1005,36	1003,81	1003,29	1002,94	1004,30	1001,19	1001,69	1000,09	1003,69	2,37	0,24	0,37
2	23	1005,95	1002,48	1002,67	1001,37	1000,13	999,75	999,28	998,01	997,08	996,32	1000,30	2,91	0,29	0,03
1	24	1006,84	1003,94	1000,80	998,63	998,72	998,27	999,35	996,59	998,18	996,08	999,74	3,33	0,33	-0,03
6	25	1006,12	1004,72	1002,24	1000,68	999,24	997,93	1000,87	998,92	996,51	995,98	1000,32	3,32	0,33	0,03
5	26	1004,25	1003,17	1001,88	1001,43	1001,11	999,18	999,20	998,55	996,83	998,53	1000,41	2,33	0,23	0,04
4	27	1006,06	1004,50	1003,53	1003,11	1003,80	1001,38	1001,45	999,98	999,22	997,37	1002,04	2,66	0,27	0,20
3	28	1006,91	1005,23	1004,57	1003,87	1002,47	1000,22	999,13	998,98	997,01	996,36	1001,48	3,64	0,36	0,15
2	29	1004,47	1002,70	1001,37	1001,39	999,66	998,70	997,37	997,64	995,25	994,65	999,32	3,20	0,32	-0,07
1	30	1006,50	1004,11	999,63	998,76	998,54	997,18	996,70	996,05	995,08	994,91	998,75	3,83	0,38	-0,13
6	31	1002,83	1001,61	1000,26	998,50	996,92	996,39	998,30	996,50	996,29	995,63	998,32	2,48	0,25	-0,17
5	32	1001,62	1001,54	1000,69	1000,92	999,38	998,45	998,29	996,61	995,93	997,47	999,09	2,06	0,21	-0,09
4	33	1004,22	1003,19	1002,51	1002,84	1001,13	1000,76	999,98	999,00	996,48	995,00	1000,51	2,98	0,30	0,05
3	34	1003,57	1002,34	1002,79	1005,04	999,96	999,44	998,79	998,39	997,41	996,38	1000,41	2,87	0,29	0,04
2	35	1002,77	1001,50	1000,49	1000,19	997,93	997,29	997,40	996,29	993,54	994,80	998,22	2,97	0,30	-0,18
1	36	1004,19	1002,33	999,18	999,35	997,72	996,89	996,70	995,07	994,39	993,34	997,92	3,44	0,34	-0,21
6	37	1003,40	1004,29	1001,15	1000,35	999,88	999,08	999,91	999,66	999,39	999,44	1000,66	1,79	0,18	0,07
5	38	1003,67	1003,70	1003,12	1002,31	1001,95	1000,40	999,67	999,06	999,63	1000,11	1001,36	1,79	0,18	0,14
4	39	1005,18	1005,74	1004,95	1004,40	1003,57	1002,89	1000,05	999,53	998,40	997,16	1002,19	3,12	0,31	0,22
3	40	1005,39	1005,09	1004,20	1003,32	1002,12	1002,95	1000,69	999,11	998,15	997,25	1001,83	2,90	0,29	0,18
2	41	1004,07	1003,56	1001,90	1001,18	998,66	998,23	997,29	998,01	995,83	996,49	999,52	2,94	0,29	-0,05
1	42	1005,66	1005,64	1002,62	1001,12	1001,31	999,55	999,06	995,26	995,58	993,44	999,92	4,21	0,42	-0,01
6	43	1003,28	1003,87	1002,06	1001,65	1002,13	999,44	1000,38	1000,68	1001,90	999,74	1001,51	1,45	0,14	0,15
5	44	1003,53	1002,83	1002,23	1002,05	1002,37	999,17	1001,25	998,66	999,18	996,73	1000,80	2,22	0,22	0,08
4	45	1003,95	1004,18	1001,98	1002,03	1004,05	998,98	1000,30	998,31	999,62	996,48	1000,99	2,67	0,27	0,10
3	46	1005,58	1005,99	1003,90	1004,22	1004,12	999,88	1000,62	999,47	999,58	999,33	1002,27	2,73	0,27	0,23
2	47	1003,71	1003,54	1001,65	1001,42	1001,01	995,78	996,96	994,89	995,31	993,59	998,79	3,85	0,39	-0,12
1	48	1006,55	1005,63	1001,79	1001,02	1002,36	997,45	1000,17	994,71	996,25	994,00	999,99	4,33	0,43	0,00
**Total**												**1001,1**	**3,13**	**0,31**	**0,11**
