# Supplementary material for: Validation of an automated system for aliquoting of HIV-1 Env-pseudotyped virus stocks
Source: PLoS One. 2018 Jan 4;13(1):e0190669. doi: 10.1371/journal.pone.0190669 (PMC5754138; doi:10.1371/journal.pone.0190669)
Supplement: S4 Table — (PDF) [file pone.0190669.s004.pdf]

S4 Table. Individual values of the 10-times measurement with the ultrasound sensors (US) of one 48-tube rack automatically aliquoted with GM containing 20% FBS plus the Average (µl), Standard Deviation (SD), Precision (%CV) and Accuracy (%Acc).

US- Sensor	Cryovial-Position	1	2	3	4	5	6	7	8	9	10	Average	SD	%CV	%Acc
6	1	996,93	997,93	998,93	999,93	1000,93	1001,93	1002,93	1003,93	1004,93	1005,93	1001,43	3,03	0,30	0,14
5	2	989,36	986,98	990,24	998,94	1004,62	1004,4	1004,99	1005,79	1006,51	1006,59	999,84	7,91	0,79	-0,02
4	3	987,35	984,37	983,96	985,03	988,71	987,37	985,93	988,66	988,09	987,84	986,73	1,77	0,18	-1,33
3	4	1004,32	997,8	995,61	992,47	991,9	990,26	992,19	991,93	991,61	991,86	994,00	4,24	0,43	-0,60
2	5	997,43	993,26	993,9	994,56	995,64	994,8	996,3	996,74	997,16	996,56	995,64	1,44	0,14	-0,44
1	6	996,97	992,62	989,06	988,26	988,58	988,25	987,94	987,74	987,21	988,13	989,48	3,02	0,31	-1,05
6	7	1002,58	1002,93	1003,67	1003,54	1003,75	1005,18	1005,6	1003,89	1003,35	1004,88	1003,94	0,98	0,10	0,39
5	8	1011,61	997,31	993,3	998,29	999,66	999,13	1001,55	1004,01	1005,88	1006,93	1001,77	5,38	0,54	0,18
4	9	1000,51	999,23	998,21	998,87	1003,14	1004,28	1003,58	1008,24	1007,94	1008,45	1003,25	3,99	0,40	0,32
3	10	999,65	1002,32	1006,61	1011,4	1010,39	1014,07	1017,02	1018,02	1016,46	1017,9	1011,38	6,62	0,65	1,14
2	11	999,92	997,62	1006,48	1006,4	1006,34	1007,29	1006,72	1007,91	1007,2	1008,37	1005,43	3,61	0,36	0,54
1	12	1010,02	1005,66	1000,17	996,83	997,07	997,63	999,93	999,64	999,1	1000,86	1000,69	4,13	0,41	0,07
6	13	993,34	992,45	993,6	994,56	994,43	991,88	991,33	991,06	991,23	992,45	992,63	1,30	0,13	-0,74
5	14	990,25	987,97	1002,55	1004,28	1007,48	1007,5	1007,33	1007,22	1008,17	1006,12	1002,89	7,48	0,75	0,29
4	15	994,85	988,11	988,42	988,59	995,88	994,23	995,24	996,25	996,72	1000,54	993,88	4,16	0,42	-0,61
3	16	1002,81	1003,31	1003,26	1006,1	1007,2	1008,03	1009,1	1011,2	1012,83	1013,2	1007,70	3,90	0,39	0,77
2	17	994,38	993,51	995,18	998,15	1000,95	1000,04	1000,92	1001,37	1002,61	1003,58	999,07	3,57	0,36	-0,09
1	18	1005,87	998,04	997,29	995,44	996,27	996,72	997,11	997,31	997,5	999,24	998,08	2,92	0,29	-0,19
6	19	995,31	993,45	992,41	991,7	991,61	991,23	992,22	992,4	992,68	993,83	992,68	1,22	0,12	-0,73
5	20	986,37	998,51	1002,31	1004,5	1008,98	1008,52	1010,89	1012,55	1013,35	1013,08	1005,91	8,46	0,84	0,59
4	21	987,55	979,29	990,57	990,08	996,17	995,37	997,38	998,3	999,15	999,29	993,32	6,42	0,65	-0,67
3	22	995,3	999,81	1003,98	1004,34	1007,48	1007,44	1010,12	1012,44	1012,61	1013,28	1006,68	5,91	0,59	0,67
2	23	996,16	993,82	995,01	996,85	998,29	999,33	1003,31	1003,45	1007,05	1007,26	1000,05	4,90	0,49	0,01
1	24	1001,21	996,09	996,06	996	997,1	997,69	997,33	998,18	998,15	998,15	997,60	1,55	0,15	-0,24
6	25	1013,68	1015,34	1016,46	1018,01	1018,08	1017,59	1017,54	1017,83	1016,95	1017,94	1016,94	1,43	0,14	1,69
5	26	1007,16	1005	1011,37	1022,93	1027,66	1028,97	1031,37	1032,27	1030,58	1031,87	1022,92	10,85	1,06	2,29
4	27	1003,93	1015,07	1016,22	1016,78	1021,91	1022,26	1021,78	1022,66	1022,87	1024,1	1018,76	6,11	0,60	1,88
3	28	1014,18	1020,06	1026,69	1028,18	1030,47	1030,98	1031,83	1034,55	1034,91	1039,66	1029,15	7,44	0,72	2,92
2	29	1026,06	1023,96	1026,7	1032,87	1032,72	1034,06	1033,46	1033,72	1033,85	1033,7	1031,11	3,90	0,38	3,11
1	30	1021,79	1024,25	1026,4	1028,77	1028,55	1028,6	1026,61	1026,49	1026,18	1025,79	1026,34	2,14	0,21	2,63
6	31	1005,29	1005,55	1007,2	1008,5	1008,27	1009,08	1009,2	1009,11	1009,4	1009,93	1008,15	1,62	0,16	0,82
5	32	1006,39	1003,09	1003,07	1002,3	1002,72	1008,87	1008,98	1008,89	1011,98	1013,47	1006,98	4,07	0,40	0,70
4	33	1005,16	1008,69	1009,56	1009,18	1008,58	1009,01	1008,36	1005,23	1005,3	1005,47	1007,45	1,89	0,19	0,75
3	34	1009,68	1009,49	1007,3	1001,43	995,02	996,34	994,95	996,76	993,61	996,71	1000,13	6,37	0,64	0,01
2	35	1023,84	1020,02	1018,97	1019,58	1018,89	1019,55	1020,3	1021,34	1022,15	1022,95	1020,76	1,73	0,17	2,08
1	36	1021,25	1021,45	1023,51	1024,62	1025,03	1024,89	1024,61	1025,23	1025,51	1025	1024,11	1,55	0,15	2,41
6	37	1013,14	1015,83	1014,27	1013,98	1014	1012,33	1011,19	1010,26	1009,78	1007,54	1012,23	2,52	0,25	1,22
5	38	1010,5	1019,63	1024,79	1025,63	1028,82	1028,84	1028,05	1028,02	1028,76	1028,29	1025,13	5,89	0,57	2,51
4	39	1006,22	1006,28	1007,4	1007,6	1011,48	1011,42	1010,71	1009,37	1009,16	1009,14	1008,88	1,96	0,19	0,89
3	40	999,26	1018,38	1026,69	1028,35	1030,52	1030,22	1030,06	1033,08	1032,17	1033,53	1026,23	10,43	1,02	2,62
2	41	1026,58	1029,46	1031,58	1031,2	1030,08	1030,81	1029,69	1030,16	1030,13	1030,7	1030,04	1,38	0,13	3,00
1	42	1035,46	1027,93	1026,35	1024,67	1022,69	1022,03	1022,34	1022,22	1022,37	1022,62	1024,87	4,23	0,41	2,49
6	43	1000,81	1002,4	1001,45	1006,64	1005,52	1005,64	1005,37	1005,66	1007,07	1006,03	1004,66	2,24	0,22	0,47
5	44	1010,12	1018,1	1019,48	1024,18	1025,04	1026,16	1024,99	1024,37	1026,85	1025,5	1022,48	5,19	0,51	2,25
4	45	1000,39	998,81	996,28	996,46	997,1	990,82	986,71	988,36	985,92	984,88	992,57	5,85	0,59	-0,74
3	46	1026,88	1019,11	1016,95	1002,36	1009,76	1009,06	1011,74	1010,41	995,93	997,27	1009,95	9,65	0,96	0,99
2	47	1014,03	1018,38	1024,39	1026,94	1023,29	1024,41	1022,21	1021,9	1022,24	1021,18	1021,90	3,57	0,35	2,19
1	48	1019,97	1020,27	1019,32	1020,51	1018,71	1018,32	1017,32	1017	1018,82	1018,44	1018,87	1,18	0,12	1,89
**Total**												**1008,2**	**13,02**	**1,29**	**0,82**
