# Supplementary material for: Validation of an automated system for aliquoting of HIV-1 Env-pseudotyped virus stocks
Source: PLoS One. 2018 Jan 4;13(1):e0190669. doi: 10.1371/journal.pone.0190669 (PMC5754138; doi:10.1371/journal.pone.0190669)
Supplement: S5 Table — (PDF) [file pone.0190669.s005.pdf]

S5 Table. Titration data of the pseudovirus (A) ZM214M.PL15 and (B) SF162.LS of automatically aliquoted pseudovirus and the manually filled virus before the automated aliquoting procedure to set the 3-fold acceptance limit and the intra-assay variability (%CV) ≤35.0%.

A

Pseudovirus	Dilution at an RLU of 150,000
ZM214M.PL15 Rack No.1 Position F8	10
ZM214M.PL15 Rack No.2 Position C5	14
ZM214M.PL15 Rack No.3 Position A1	11
ZM214M.PL15 before automated aliquoting	11
Acceptance limit 3-fold range	4 to 33
Average	12
SD	1.73
%CV	15.1

B

Pseudovirus	RLU
SF162.LS Rack No.1 Position F5	67,068
SF162.LS Rack No.6 Position F5	73,062
SF162.LS Rack No.12 Position F5	69,538
SF162.LS before automated aliquoting	77,942
Acceptance limit 3-fold range	25,981 to 233,826
Average	71,903
SD	4.72
%CV	6.56
