# Supplementary material for: Validation of an automated system for aliquoting of HIV-1 Env-pseudotyped virus stocks
Source: PLoS One. 2018 Jan 4;13(1):e0190669. doi: 10.1371/journal.pone.0190669 (PMC5754138; doi:10.1371/journal.pone.0190669)
Supplement: S6 Table — (PDF) [file pone.0190669.s006.pdf]

S6 Table. Titration data of the pseudovirus SF162.LS of (A) Batch #1 and (B) Batch #2 to set the acceptance limit for the intra-assay variability (%CV) ≤35.0%.

A

**Virus**	**Intra-Assay Variability Operator 1**<br>Dilution at 150,000 RLU	**Intra-Assay Variability Operator 2**<br>Dilution at 150,000 RLU
SF162.LS Batch #1	10	9
SF162.LS Batch #1	9	18
SF162.LS Batch #1	9	9
SF162.LS Batch #1	9	12
SF162.LS Batch #1	9	9
SF162.LS Batch #1	10	9
SF162.LS Batch #1	12	9
SF162.LS Batch #1	10	9
Average	10	11
SD	1,04	3,21
%CV	10,6	30,5

B

**Virus**	**Intra-Assay Variability Operator 1**<br>Dilution at 150,000 RLU	**Intra-Assay Variability Operator 2**<br>Dilution at 150,000 RLU
SF162.LS Batch #2	6	6
SF162.LS Batch #2	6	7
SF162.LS Batch #2	5	7
SF162.LS Batch #2	5	7
SF162.LS Batch #2	5	7
SF162.LS Batch #2	5	6
SF162.LS Batch #2	7	6
SF162.LS Batch #2	7	5
SF162.LS Batch #2	7	8
Average	6	7
SD	0,93	0,83
%CV	15,8	12,7
