# Supplementary material for: Validation of an automated system for aliquoting of HIV-1 Env-pseudotyped virus stocks
Source: PLoS One. 2018 Jan 4;13(1):e0190669. doi: 10.1371/journal.pone.0190669 (PMC5754138; doi:10.1371/journal.pone.0190669)
Supplement: S7 Table — Compared are the neutralization titers of the automatically and the manually aliquoted reference viruses by assaying five defined test reagents. (PDF) [file pone.0190669.s007.pdf]

S7 Table. Parallel performed neutralization assays to determine the acceptance limit to verify the integrity/quality of the automatically aliquoted HIV-1 pseudovirus stocks. Compared are the neutralization titers of the automatically and the manually aliquoted reference viruses by assaying five defined test reagents.

	IC50 values (µg/ml) of virus stocks determined with HIV-1 neutralizing test reagents				
Pseudovirus	sCD4	IgG1b12	2F5	4E10	TriMab
ZM214M.PL15 (Rack No. 3)	10.22	4.62	>25.00	30.63	12.95
ZM214M.PL15 (manual reference stock)	7.63	4.44	>25.00	15.57	10.10
acceptance limit	2.54 to 22.89	1.48 to 13.32	>25.00	5.19 to 46.71	3.37 to 30.30
SF162.LS (Rack No. 10)	0.18	0.05	3.73	6.97	0.12
SF162.LS.7 (manual reference stock)	0.18	0.05	4.92	11.12	0.15
acceptance limit	0.06 to 0.54	0.02 to 0.15	1.64 to 14.76	3.71 to 33.36	0.05 to 0.45
