# Supplementary material for: Validation of an automated system for aliquoting of HIV-1 Env-pseudotyped virus stocks
Source: PLoS One. 2018 Jan 4;13(1):e0190669. doi: 10.1371/journal.pone.0190669 (PMC5754138; doi:10.1371/journal.pone.0190669)
Supplement: S8 Table — (PDF) [file pone.0190669.s008.pdf]

**S8 Table. Titration data of the pseudovirus (A) CH110.2 and (B) Q842.d12 incubated under different conditions before storage at -80°C.**

**A**

| Pseudovirus | Dilution at an RLU of 150,000 |
|---|---|
| CH110.2 directly frozen at -80°C | 150 |
| CH110.2 incubation 2 hrs at RT | 160 |
| CH110.2 incubation 6 hrs at RT | 150 |
| CH110.2 incubation 4 hrs at 4°C followed by 2 hrs at RT | 130 |

**B**

| Pseudovirus | Dilution at an RLU of 150,000 |
|---|---|
| Q842.d12 directly frozen at -80°C | 45 |
| Q842.d12 incubation 2 hrs at RT | 45 |
| Q842.d12 incubation 6 hrs at RT | 45 |
| Q842.d12 incubation 4 hrs at 4°C followed by 2 hrs at RT | 40 |
