# Supplementary material for: Validation of an automated system for aliquoting of HIV-1 Env-pseudotyped virus stocks
Source: PLoS One. 2018 Jan 4;13(1):e0190669. doi: 10.1371/journal.pone.0190669 (PMC5754138; doi:10.1371/journal.pone.0190669)
Supplement: S9 Table — (PDF) [file pone.0190669.s009.pdf]

S9 Table. Individual results of the validation experiment of the gravimetrical measurement for the selected volume of 100 µl plus the Average (µl), Standard Deviation (SD), Precision (%CV) and Accuracy (%Acc).

	Channel 1	Channel 2	Channel 3	Channel 4	Channel 5	Channel 6	Channel 7	Channel 8	Average	SD	%CV
1	97,458	96,830	97,090	97,450	94,422	96,610	96,512	97,232	96,7	1,0	1,0
2	97,160	97,082	96,870	97,754	96,940	96,680	96,008	97,010	96,9	0,5	0,5
3	96,764	96,792	96,970	96,746	96,878	96,676	96,282	96,900	96,8	0,2	0,2
4	96,902	96,810	96,762	96,516	96,604	96,808	95,950	96,852	96,7	0,3	0,3
5	96,826	96,760	96,562	97,716	96,658	96,180	95,980	97,014	96,7	0,5	0,5
6	97,012	96,936	96,480	97,114	96,690	96,642	96,670	97,076	96,8	0,2	0,2
7	96,896	96,626	96,416	97,154	96,626	96,936	95,872	96,900	96,7	0,4	0,4
8	96,632	96,482	96,304	96,712	96,930	96,634	95,700	96,872	96,5	0,4	0,4
9	96,620	96,300	96,488	96,252	96,534	96,532	95,878	96,966	96,4	0,3	0,3
10	96,526	96,288	96,306	96,962	96,510	96,700	96,734	96,832	96,6	0,2	0,3
11	96,682	96,234	96,222	96,160	97,180	95,994	96,476	96,758	96,5	0,4	0,4
12	96,518	96,298	95,886	96,846	96,334	95,884	96,756	96,794	96,4	0,4	0,4
Average	96,8	96,6	96,5	96,9	96,5	96,5	96,2	96,9		**Overall**	
SD	0,3	0,3	0,3	0,5	0,7	0,3	0,4	0,1	**Average**	**SD**	**%CV**
%CV	0,3	0,3	0,4	0,5	0,7	0,3	0,4	0,1	**96,6**	**0,45**	**0,47**

**%Acc**	**-3,4**
**Min**	**94,4**
**Max**	**97,8**
