# Supplementary material for: Validation of an automated system for aliquoting of HIV-1 Env-pseudotyped virus stocks
Source: PLoS One. 2018 Jan 4;13(1):e0190669. doi: 10.1371/journal.pone.0190669 (PMC5754138; doi:10.1371/journal.pone.0190669)
Supplement: S11 Table — (PDF) [file pone.0190669.s011.pdf]

S11 Table. Individual values of the 10-times measurement with the ultrasound sensors (US) of one 48-tube rack automatically aliquoted with GM containing 20% FBS plus the Average (µl), Standard Deviation (SD), Precision (%CV) and Accuracy (%Acc).

US- Sensor	Cryovial-Position	1	2	3	4	5	6	7	8	9	10	Average	SD	%CV	%Acc
6	1	1000,51	1000,08	998,69	999,7	999,16	996,94	995,36	994,8	996,8	1003,37	998,54	2,60	0,26	-0,15
5	2	1001,05	1000,81	998,06	994,7	994,34	990,7	989,75	991,08	1001,72	1002,83	996,50	5,01	0,50	-0,35
4	3	983,48	983,81	982,82	983,1	984,47	984,22	984,18	985,99	988,65	991,23	985,20	2,72	0,28	-1,48
3	4	997,16	993,43	986,97	981,27	973,89	972,68	976,43	973,69	992,27	998,74	984,65	10,29	1,05	-1,53
2	5	996,1	993,52	993,11	991,91	995,79	989,28	987,16	985,33	987,54	994,79	991,45	3,87	0,39	-0,85
1	6	985,43	983,77	983,13	983,79	985,1	984,88	985,37	989,47	991,97	996,46	986,94	4,34	0,44	-1,31
6	7	998,3	998,97	999,05	998,01	997,82	997,15	993,98	993,93	997,01	1001,93	997,62	2,37	0,24	-0,24
5	8	992,25	992,11	989,93	988,12	988,79	989,55	987,42	993,41	996,18	1001,24	991,90	4,24	0,43	-0,81
4	9	997,59	997,16	995,84	992,26	991,34	989,37	988,01	989,18	996,58	1001,03	993,84	4,38	0,44	-0,62
3	10	1001,72	999,72	995,43	987,81	979,89	973,83	980,44	977,25	985,17	997,22	987,85	10,07	1,02	-1,22
2	11	987,79	985,92	986,19	984,82	987,53	985,71	983,25	984,02	985,78	995,11	986,61	3,30	0,33	-1,34
1	12	991,62	991,1	991,7	990,51	990,59	991,44	992,73	997,44	998,28	1002,08	993,75	4,03	0,41	-0,63
6	13	999,66	999,59	999,6	999,14	998,62	998,92	996,81	995,92	999,4	1003,07	999,07	1,89	0,19	-0,09
5	14	998,95	989,96	985,45	985,04	985,54	983,18	982,83	985,19	988,38	992,62	987,71	4,98	0,50	-1,23
4	15	982,51	976,4	973,45	973,7	976,49	978,56	981,71	985,37	987,79	991,17	980,72	6,04	0,62	-1,93
3	16	1005,13	1004,09	1003,95	1002,25	1002	1000,12	987,34	986,5	983,66	992,83	996,79	8,34	0,84	-0,32
2	17	977,17	975	974,98	975,28	979,62	978,89	979,02	981,43	983,95	986,3	979,16	3,85	0,39	-2,08
1	18	979,78	978,82	979,01	978,05	979,21	982,54	984,64	985,26	986,06	991	982,44	4,23	0,43	-1,76
6	19	992,69	992,15	992,72	987,99	986,24	984,85	983,9	983,43	985,28	993,55	988,28	4,08	0,41	-1,17
5	20	993,22	988,8	985,84	983,74	981,03	978	980,12	980,32	990,7	992,96	985,47	5,66	0,57	-1,45
4	21	984,72	983,47	981,9	981,34	980,35	980,12	980,31	967,05	966,53	967,03	977,28	7,33	0,75	-2,27
3	22	1006,37	1005,39	1001,09	999,78	997,94	994,95	975,73	976,68	977,57	988,87	992,44	11,97	1,21	-0,76
2	23	969,78	969,26	969,28	967,43	968,55	969,71	969,45	969,42	677,44	974,62	940,49	92,45	9,83	-5,95
1	24	978,86	978,84	978,42	978,27	980,46	983,58	984,23	984,43	986,95	990,21	982,43	4,12	0,42	-1,76
6	25	1019,78	1019,46	1017,71	1017,22	1016,8	1015,51	1014,89	1012,48	1012,21	1019,27	1016,53	2,74	0,27	1,65
5	26	1028,19	1027,51	1026,85	1027,15	1026,27	1021,09	1017,2	1013,25	1004,84	1013,64	1020,60	8,04	0,79	2,06
4	27	1011,19	1009,47	1011,11	1008,93	1008,47	1007,72	1007,79	1001,71	992,99	1002,55	1006,19	5,63	0,56	0,62
3	28	1022,25	1017,84	1017,67	1015,92	1015,15	1011,28	1010,2	1007,99	1004,58	1016,64	1013,95	5,32	0,52	1,40
2	29	1008,68	1006,65	1007,07	1003,42	1004,32	1006,93	1008,66	1008,3	1011,45	1015,39	1008,09	3,43	0,34	0,81
1	30	1000,51	1000,64	1000,06	1000,22	1001,33	1001,2	1004,88	1004,47	1007,3	1010,92	1003,15	3,66	0,37	0,32
6	31	1017,84	1017,32	1016,83	1016,7	1015,8	1015,74	1013,55	1009,95	1009,59	1009,08	1014,24	3,45	0,34	1,42
5	32	1016,16	1014,34	1012,3	1010,91	1006,4	1002	998,17	997,79	1003,84	1005,64	1006,76	6,50	0,65	0,68
4	33	991,11	990,17	987,79	986,41	986,06	985,44	967,81	968,89	984,08	984,21	983,20	8,16	0,83	-1,68
3	34	997,6	989,01	987,27	989,24	986,46	988,44	976,76	977,32	996,1	996,37	988,46	7,24	0,73	-1,15
2	35	1008,73	1007,62	1008,75	1005,78	1006,62	1009,04	1008,66	995,32	1000,26	1006,75	1005,75	4,49	0,45	0,58
1	36	994,36	992,06	993,55	994	994,8	994,8	996,75	997,39	999,85	1002,83	996,04	3,25	0,33	-0,40
6	37	1012,05	1012,12	1010,99	1009,8	1009,79	1008,28	1009,31	1007,56	1011,69	1012,14	1010,37	1,67	0,17	1,04
5	38	1001,1	1001,07	999,68	1001,93	996,88	999,11	998,29	1001,56	1010,5	1008,33	1001,85	4,32	0,43	0,18
4	39	996,83	996,81	996,84	995,46	995,86	995,26	995,98	993,63	993,81	980,76	994,12	4,83	0,49	-0,59
3	40	1017,28	1012,32	1010,59	1003,69	1004,98	987,41	979,72	951,75	994,45	988,95	995,11	19,47	1,96	-0,49
2	41	1008,34	1006,85	1006,05	1004,31	1004,79	1008,9	1008,5	1009,31	1005,95	999,52	1006,25	2,93	0,29	0,63
1	42	996,61	995,04	995,45	994,44	995,18	994,95	999,37	999,76	1003,4	1004,98	997,92	3,80	0,38	-0,21
6	43	1000,99	1000,83	1000,16	999,24	1000,6	1000,49	1000,25	998,93	1005,31	1009,64	1001,64	3,30	0,33	0,16
5	44	999,16	997,99	999,69	999,12	999,3	1000,72	1001,29	996,92	1007,31	1008,08	1000,96	3,76	0,38	0,10
4	45	975,41	970,19	970,33	966,13	966,65	965,71	966,67	940,95	969,91	970,53	966,25	9,36	0,97	-3,38
3	46	1000,52	991,73	987,22	983,9	980,76	976,26	958,65	927,72	931,22	944,89	968,29	25,97	2,68	-3,17
2	47	981,19	978,11	978,77	978,81	976,88	983,53	983,46	982,69	986,54	987,83	981,78	3,68	0,37	-1,82
1	48	996,09	993,68	994,74	991,4	991,09	990,09	992,8	985,63	988,22	975,83	989,96	5,84	0,59	-1,00
**Total**												**993,0**	**20,06**	**2,02**	**-0,70**
