# Supplementary material for: Validation of an automated system for aliquoting of HIV-1 Env-pseudotyped virus stocks
Source: PLoS One. 2018 Jan 4;13(1):e0190669. doi: 10.1371/journal.pone.0190669 (PMC5754138; doi:10.1371/journal.pone.0190669)
Supplement: S12 Table — (PDF) [file pone.0190669.s012.pdf]

S12 Table. Individual values of the 10-times measurement with the ultrasound sensors (US) of one 48-tube rack automatically aliquoted with GM containing 20% FBS plus the Average (µl), Standard Deviation (SD), Precision (%CV) and Accuracy (%Acc).

US- Sensor	Cryovial-Position	1	2	3	4	5	6	7	8	9	10	Average	SD	%CV	%Acc
6	1	995,96	995,72	995,18	995,52	994,94	995,38	997,62	999,84	1000,34	1007,22	997,77	3,85	0,39	-0,22
5	2	1009,17	1008,82	1008,25	1007,34	1005,74	1002,7	998,05	996,57	989,89	997,48	1002,40	6,59	0,66	0,24
4	3	990,35	990	989,45	989,83	989,88	989,36	989,41	988,87	989,06	996,64	990,29	2,28	0,23	-0,97
3	4	1008,76	1004,34	1007,06	1008,28	1003,04	1002,16	1000,83	1001,35	1001,88	1004,95	1004,27	2,92	0,29	0,43
2	5	998,42	997,02	994,66	993,21	992,99	992,96	994,6	995,95	997,32	1000,66	995,78	2,58	0,26	-0,42
1	6	996,18	992,25	992	991,31	992,75	993,18	993,47	993,59	995,5	1004,23	994,45	3,75	0,38	-0,56
6	7	1007,66	1007,43	1008,5	1008,26	1007,28	1007,35	1007,44	1007,46	1008,43	1009,95	1007,98	0,84	0,08	0,80
5	8	1019,32	1019,5	1019,38	1019,38	1018,72	1018,84	1018,46	1019,07	1000,82	1007,57	1016,11	6,48	0,64	1,61
4	9	1008,7	1008,7	1008,97	1008,16	1007,76	1007,26	1006,18	1002,98	1001,73	1001,35	1006,18	3,01	0,30	0,62
3	10	1008,2	1007,81	1007,7	1010,26	1011,35	1011,92	1012,12	1011,8	1014,02	1007,71	1010,29	2,29	0,23	1,03
2	11	991,5	989,56	988,97	989,51	989,01	988,98	990,38	993,31	993,88	999,57	991,47	3,37	0,34	-0,85
1	12	1003,35	1003,54	1004,23	1003,4	1003,5	1004,13	1004,23	1005,03	1004,96	1012,42	1004,88	2,72	0,27	0,49
6	13	1003,39	1003,47	1003,68	1002,62	1002,95	1001,78	1000,89	1000,01	999,58	1007,89	1002,63	2,36	0,24	0,26
5	14	1014,26	1013,77	1014,31	1013	1013,55	1013,94	1012,51	999,28	996,37	999,95	1009,09	7,36	0,73	0,91
4	15	995,79	995,74	994,79	995,01	994,67	995,23	993,04	992,24	994,56	995,84	994,69	1,19	0,12	-0,53
3	16	1009,12	1007,57	1006,2	1007,35	1008,57	1009,48	1003,82	1002,04	1004,07	1007,55	1006,58	2,49	0,25	0,66
2	17	1000,87	1001,02	999,23	997,39	993,49	993,99	991,67	987,8	987,12	992,89	994,55	5,00	0,50	-0,55
1	18	991,42	991,31	988,54	988,32	986,87	988,65	987,79	988,82	988,99	999,13	989,98	3,51	0,35	-1,00
6	19	999,82	1002,65	1002,69	1003,25	1001,04	998,79	996,99	996,81	997,53	1003,92	1000,35	2,73	0,27	0,03
5	20	1002,78	1006,59	1006,24	1014,63	1013,28	1014,95	1014,17	1011,41	989,47	986,45	1006,00	10,38	1,03	0,60
4	21	995,97	996,1	995,3	995,44	995,31	994,56	993,54	994,1	987,66	995,95	994,39	2,51	0,25	-0,56
3	22	1000,11	1001,41	1004,34	1007,44	1006,96	1007,45	1007,71	1007,83	1008,38	1000,75	1005,24	3,29	0,33	0,52
2	23	994,28	997,29	996,63	999,96	989,35	988,52	987,21	985,17	984,62	985,23	990,83	5,71	0,58	-0,92
1	24	993,76	993,7	992,67	992,5	991,1	992,47	986,16	989,36	990,54	997,58	991,98	3,02	0,30	-0,80
6	25	1022,08	1021,39	1022,71	1022,82	1022,68	1023,83	1023,77	1022,67	1018,79	1018,75	1021,95	1,82	0,18	2,19
5	26	1028,14	1028,09	1027,27	1034,28	1032,97	1033,41	1033,05	1033,5	1029,45	1018,53	1029,87	4,79	0,47	2,99
4	27	1016,18	1016,15	1015,99	1014,1	1013,08	1011,26	1011,33	1011,02	1011,45	1010,51	1013,11	2,32	0,23	1,31
3	28	1009,95	1008,27	1012,93	1012,99	1018,75	1020,43	1025,95	1030,72	1031,21	1018,18	1018,94	8,22	0,81	1,89
2	29	1021,92	1022,32	1021,46	1022,08	1019,72	1017,96	1018,41	1016,48	1015,17	1019,17	1019,47	2,49	0,24	1,95
1	30	1012,71	1012,76	1014,53	1014,23	1012,22	1013,73	1013,48	1013,38	1013,47	1020,57	1014,11	2,37	0,23	1,41
6	31	1016,47	1019,64	1018,9	1019,49	1019,36	1017,69	1018,22	1017,75	1016,41	1017,54	1018,15	1,19	0,12	1,81
5	32	1017,68	1020,93	1022,84	1023,12	1022,87	1023,54	1021,5	1022,6	1021,37	1005,71	1020,22	5,37	0,53	2,02
4	33	993,97	999,58	1001,04	1000,7	1003,67	1001,72	1003,21	1003,82	1002,63	999,88	1001,02	2,90	0,29	0,10
3	34	1008,46	1009,44	1013,2	1016,09	1017,48	1018,59	1018,66	1016,2	1014,99	999,83	1013,29	5,90	0,58	1,33
2	35	1009,61	1010,76	1011,05	1012,69	1012,72	1014,23	1017,23	1015,13	1012,17	1011,9	1012,75	2,25	0,22	1,27
1	36	1007,21	1007,02	1008,02	1006,2	1005,08	1004,1	1002,73	1001,66	1002,22	1009,73	1005,40	2,69	0,27	0,54
6	37	1010,07	1012,38	1012,78	1012,83	1018,72	1019,46	1016,04	1014,66	1014,05	1011,78	1014,28	3,01	0,30	1,43
5	38	1016,19	1018,3	1019,53	1018,9	1016,88	1027,66	1027,73	1026,09	1019,73	1006,92	1019,79	6,28	0,62	1,98
4	39	996,89	995,41	997,8	997,16	1000,01	1002,51	1006,66	1006,86	1006,02	1004,83	1001,42	4,48	0,45	0,14
3	40	979,06	984,75	989,3	976,45	993,1	1001,05	1006,06	998,62	1017,21	1021,98	996,76	15,28	1,53	-0,32
2	41	1007,74	1012,8	1012,84	1012,57	1011,74	1014,77	1016,68	1015,7	1015,9	1013,05	1013,38	2,59	0,26	1,34
1	42	1001,76	1003,22	1004,78	1004,25	1004,24	1006,52	1006,25	1010,69	1012,25	1010,6	1006,46	3,56	0,35	0,65
6	43	989,12	988,28	995,84	998,07	998,16	1000,46	1009,75	1012,37	1014,01	1009,91	1001,60	9,41	0,94	0,16
5	44	1000,7	1007,9	1006,87	1003,9	1004,12	1006,8	1009,36	1004,71	991,89	1017,79	1005,40	6,57	0,65	0,54
4	45	991,34	993,6	994,8	989,8	993,9	998,47	999,18	998,89	1000,2	993,58	995,38	3,59	0,36	-0,46
3	46	983,56	1004,59	1008,64	1010,13	1008,69	1011,69	1013,48	1017,39	1019,73	1020,74	1009,86	10,60	1,05	0,99
2	47	1004,51	1005,51	1004,58	1005,19	1002,82	1003,8	1004,8	1004,34	1001,68	990,05	1002,73	4,60	0,46	0,27
1	48	1000,41	1001,73	999,5	995,79	993,56	995,39	994,3	990,6	997,92	1001,82	997,10	3,78	0,38	-0,29
**Total**												**1005,4**	**10,79**	**1,07**	**0,54**
