# Supplementary material for: Validation of an automated system for aliquoting of HIV-1 Env-pseudotyped virus stocks
Source: PLoS One. 2018 Jan 4;13(1):e0190669. doi: 10.1371/journal.pone.0190669 (PMC5754138; doi:10.1371/journal.pone.0190669)
Supplement: S13 Table — (PDF) [file pone.0190669.s013.pdf]

S13 Table. Individual values of the 10-times measurement with the ultrasound sensors (US) of one 48-tube rack manually aliquoted with GM containing 20% FBS plus the Average (µl), Standard Deviation (SD), Precision (%CV) and Accuracy (%Acc).

US- Sensor	Cryovial-Position	1	2	3	4	5	6	7	8	9	10	Average	SD	%CV	%Acc
6	1	921,17	920,02	921,27	924,7	924,92	926,32	925,42	927,82	927,3	928,94	924,79	3,05	0,33	-7,52
5	2	920,82	923,34	927,74	929,1	927,89	927,78	929,07	929,8	930,16	932,26	927,80	3,36	0,36	-7,22
4	3	889,19	888,88	893,46	899,01	910,19	897,13	907,95	907,22	907,11	913,18	901,33	8,93	0,99	-9,87
3	4	890,81	894,41	895,04	884,91	885,07	889,02	884,51	882,04	876,95	880,73	886,35	5,90	0,67	-11,37
2	5	911,28	911,8	913,11	914,62	911,78	914,06	915,61	918,53	916,98	917	914,48	2,52	0,28	-8,55
1	6	918,02	920,39	917,17	921,2	922,27	923,91	922,85	925,64	922,34	923,34	921,71	2,61	0,28	-7,83
6	7	914,69	922,84	928,56	929,07	928,8	929,45	929,9	931,88	933,06	931,66	927,99	5,43	0,59	-7,20
5	8	917,74	923,46	926,8	927,44	928,51	929,38	929,85	930,42	928,73	931,53	927,39	4,06	0,44	-7,26
4	9	919,19	917,31	921,78	927,08	921,61	929,1	928,01	926,78	921,91	926,79	923,96	4,08	0,44	-7,60
3	10	889,89	878,4	854,3	877,02	856,14	856,24	876,56	868,67	851,04	866,22	867,45	12,90	1,49	-13,26
2	11	907,49	908,47	909,53	907,47	908,17	909,04	908,79	909,52	909,85	910,47	908,88	1,00	0,11	-9,11
1	12	922,12	920,7	920,3	920,95	923,91	924,15	926,91	927,32	923,98	924,64	923,50	2,47	0,27	-7,65
6	13	901,57	904,44	908,7	913,56	912,93	905,94	913,07	918,72	913,67	917,53	911,01	5,64	0,62	-8,90
5	14	920,52	920,26	927,72	924,16	924,11	926,11	930,08	926,11	930,05	927,15	925,63	3,43	0,37	-7,44
4	15	895,19	899,88	913,73	918,28	924,46	917,19	923,78	923,5	921,01	920,1	915,71	10,19	1,11	-8,43
3	16	899,41	887,92	890,97	887,92	886,92	885,83	889,99	888,74	887,49	886,55	889,17	3,92	0,44	-11,08
2	17	908,57	906,88	906,4	903,35	901,97	903,79	902,63	905,43	903,14	905,48	904,76	2,12	0,23	-9,52
1	18	918,18	924,63	926,23	926,05	927,6	928,63	930,46	929,62	928,31	930,49	927,02	3,66	0,39	-7,30
6	19	900,77	913,93	920,24	922,71	924,38	925,12	925,1	928,3	928,39	929,33	921,83	8,68	0,94	-7,82
5	20	922,54	922,59	928,86	926,92	926,28	927,14	928,45	926,32	928,77	925,7	926,36	2,28	0,25	-7,36
4	21	888,73	896,07	899,13	918,64	916,33	910,85	925,37	918,91	919,8	910,22	910,41	11,97	1,32	-8,96
3	22	871,85	869,43	871,46	845,68	845,77	850,55	850,75	864,2	856,07	845,52	857,13	11,07	1,29	-14,29
2	23	924,27	932,99	934,77	934,38	933,98	935,93	936,55	937,04	937,91	936,1	934,39	3,86	0,41	-6,56
1	24	909,88	912,18	916,74	918,35	918,84	920,41	922,71	920,08	921,12	920,5	918,08	4,09	0,45	-8,19
6	25	687,28	885,21	892,95	893,47	893,07	893,28	891,59	897,61	896,76	896,87	872,81	65,28	7,48	-12,72
5	26	910,68	911,12	914,11	912,62	913,92	914,32	913,72	915,64	916,97	914,46	913,76	1,90	0,21	-8,62
4	27	885,79	886,79	886,77	894,05	893,54	895,63	894,31	895,23	897,97	900,81	893,09	5,06	0,57	-10,69
3	28	884,32	894,56	898,26	900,52	897,35	902,11	899	901,37	902,46	902,02	898,20	5,48	0,61	-10,18
2	29	916	920,49	928,76	928,2	926,47	929,34	931,44	927,96	933,7	932,13	927,45	5,41	0,58	-7,26
1	30	912,44	911,28	912,94	913,98	918,12	921,09	916,54	922,69	918,24	919,52	916,68	3,90	0,42	-8,33
6	31	919,6	918,24	912,09	916,61	920,11	923,48	926,47	919,5	927,09	918,07	920,13	4,54	0,49	-7,99
5	32	912,52	913,2	916,46	917,98	918,54	918,37	918,67	920,73	920,04	921,05	917,76	2,92	0,32	-8,22
4	33	890,79	892,32	891,01	896,77	896,25	897,11	896,01	897,6	897,84	897,51	895,32	2,81	0,31	-10,47
3	34	887,51	895,27	889,07	879,88	879,36	894,07	894,76	887,76	887,05	894,38	888,91	5,88	0,66	-11,11
2	35	917,4	919,4	923,16	923,99	921,7	924,85	923,02	923,91	926,17	924,21	922,78	2,63	0,29	-7,72
1	36	907,3	910,75	909,62	913,28	914	915,3	914,05	917,04	916,4	916,86	913,46	3,28	0,36	-8,65
6	37	910,21	909,78	904,04	908,9	908,9	908,95	908,15	907,71	908,92	911,63	908,72	1,98	0,22	-9,13
5	38	917,93	917,33	918,85	921,82	921,58	921,57	923,15	924,19	921,52	926,25	921,42	2,78	0,30	-7,86
4	39	906,81	900,85	899,66	902,07	907,61	900,69	902,56	900,45	900,21	904,17	902,51	2,81	0,31	-9,75
3	40	871,66	864,97	843,4	871,45	829,59	844,64	867,86	856,4	853,03	867,33	857,03	14,21	1,66	-14,30
2	41	922,27	919,35	924,18	925,63	928,44	930,51	932,14	932,18	932,72	932,08	927,95	4,81	0,52	-7,21
1	42	921,33	924,25	922,18	923,81	923,92	922,99	925,78	925,21	927,48	923,15	924,01	1,80	0,19	-7,60
6	43	903,57	902,07	902,54	901,95	904,45	901,66	903,39	905,73	903,12	902	903,05	1,29	0,14	-9,70
5	44	916,84	917,6	916,71	922,37	924,59	924,5	923,64	922,42	921,44	924,06	921,42	3,18	0,35	-7,86
4	45	878,78	880,76	885,65	886,05	884,79	887,12	884,04	884,54	884,61	887,67	884,40	2,74	0,31	-11,56
3	46	874,04	867,46	839,04	847,17	858,75	833,36	862,17	842,9	844,65	840,71	851,03	13,61	1,60	-14,90
2	47	899,46	906,56	919,12	918,22	918,22	918,36	919,64	921,73	919,23	911,87	915,24	7,12	0,78	-8,48
1	48	907,29	905,72	905,42	907,11	906,26	906,46	906,95	907,99	907,48	905,35	906,60	0,91	0,10	-9,34
**Total**												**908,4**	**22,96**	**2,53**	**-9,16**
