# Supplementary material for: Validation of an automated system for aliquoting of HIV-1 Env-pseudotyped virus stocks
Source: PLoS One. 2018 Jan 4;13(1):e0190669. doi: 10.1371/journal.pone.0190669 (PMC5754138; doi:10.1371/journal.pone.0190669)
Supplement: S14 Table — Shown are the results of three 48-well plates (A, B and C), whereby each channel distributed 6 times 500 μl distilled water and 500 μl Orange G. (PDF) [file pone.0190669.s014.pdf]

S14 Table. Average OD, Standard Deviation (SD) and precision (%CV) of the photometric test.
Shown are the results of three 48-well plates (A, B and C), whereby each channel distributed 6 times 500 µl distilled water and 500 µl Orange G.

A

	Channel 1	Channel 2	Channel 3	Channel 4	Channel 5	Channel 5	Channel 6	Channel 7	Average	SD	%CV
1	1,365	1,374	1,355	1,353	1,391	1,377	1,382	1,373	1,371	0,013	0,9
2	1,363	1,365	1,354	1,341	1,380	1,378	1,385	1,378	1,368	0,015	1,1
3	1,361	1,368	1,356	1,350	1,378	1,376	1,386	1,374	1,369	0,012	0,9
4	1,360	1,356	1,348	1,351	1,389	1,364	1,376	1,382	1,366	0,015	1,1
5	1,350	1,360	1,340	1,340	1,367	1,375	1,381	1,363	1,359	0,015	1,1
6	1,351	1,344	1,335	1,330	1,369	1,362	1,348	1,372	1,351	0,015	1,1
Average	1,358	1,361	1,348	1,344	1,379	1,372	1,376	1,374	Overall		
SD	0,006	0,010	0,009	0,009	0,010	0,007	0,014	0,007	Average	SD	%CV
%CV	0,4	0,8	0,6	0,7	0,7	0,5	1,0	0,5	1,364	0,015	1,1

B

	Channel 1	Channel 2	Channel 3	Channel 4	Channel 5	Channel 5	Channel 6	Channel 7	Average	SD	%CV
1	1,355	1,357	1,353	1,352	1,390	1,386	1,398	1,381	1,372	0,019	1,4
2	1,356	1,366	1,346	1,345	1,379	1,367	1,391	1,374	1,366	0,016	1,2
3	1,357	1,353	1,345	1,346	1,377	1,366	1,378	1,377	1,362	0,014	1,0
4	1,360	1,355	1,340	1,341	1,367	1,373	1,375	1,378	1,361	0,015	1,1
5	1,349	1,351	1,343	1,331	1,369	1,365	1,364	1,366	1,355	0,014	1,0
6	1,343	1,341	1,340	1,331	1,360	1,356	1,367	1,357	1,350	0,013	0,9
Average	1,353	1,354	1,344	1,341	1,374	1,369	1,379	1,372	Overall		
SD	0,006	0,008	0,005	0,009	0,011	0,010	0,013	0,009	Average	SD	%CV
%CV	0,5	0,6	0,4	0,7	0,8	0,7	1,0	0,6	1,361	0,016	1,2

C

	Channel 1	Channel 2	Channel 3	Channel 4	Channel 5	Channel 5	Channel 6	Channel 7	Average	SD	%CV
1	1,363	1,365	1,357	1,358	1,394	1,385	1,400	1,383	1,376	0,017	1,2
2	1,365	1,367	1,352	1,350	1,383	1,374	1,391	1,375	1,370	0,014	1,0
3	1,360	1,356	1,349	1,351	1,379	1,369	1,377	1,378	1,365	0,013	0,9
4	1,365	1,363	1,340	1,345	1,367	1,374	1,375	1,378	1,363	0,014	1,0
5	1,363	1,358	1,349	1,336	1,375	1,368	1,369	1,369	1,361	0,013	0,9
6	1,340	1,336	1,337	1,330	1,361	1,354	1,368	1,359	1,348	0,014	1,0
Average	1,360	1,357	1,347	1,345	1,376	1,371	1,380	1,374	Overall		
SD	0,010	0,011	0,007	0,010	0,012	0,010	0,013	0,009	Average	SD	%CV
%CV	0,7	0,8	0,5	0,8	0,9	0,7	0,9	0,6	1,364	0,016	1,2
