# Supplementary material for: Validation of an automated system for aliquoting of HIV-1 Env-pseudotyped virus stocks
Source: PLoS One. 2018 Jan 4;13(1):e0190669. doi: 10.1371/journal.pone.0190669 (PMC5754138; doi:10.1371/journal.pone.0190669)
Supplement: S15 Table — (PDF) [file pone.0190669.s015.pdf]

S15 Table. Intermediate precision after 10-times measurement with the ultrasound sensors (US) of one rack automatically aliquoted.

Average Volume Measurement 1 (µl)	Average Volume Measurement 2 (µl)	Average Volume Measurement 3 (µl)	Mean	Standard Deviation	Intermediate precision (%CV)
1007	993,0	1005,4	1001,8	7,66	0,76
