# Supplementary material for: Validation of an automated system for aliquoting of HIV-1 Env-pseudotyped virus stocks
Source: PLoS One. 2018 Jan 4;13(1):e0190669. doi: 10.1371/journal.pone.0190669 (PMC5754138; doi:10.1371/journal.pone.0190669)
Supplement: S16 Table — Compared are the neutralization titers of the automatically and the manually aliquoted historical reference viruses by assaying five defined test reagents with the defined 3-fold acceptance limit. (PDF) [file pone.0190669.s016.pdf]

S16 Table. Parallel performed neutralization assays to verify the integrity of the large-scale prepared virus stock PVO.4 after the automated aliquoting process. Compared are the neutralization titers of the automatically and the manually aliquoted historical reference viruses by assaying five defined test reagents with the defined 3-fold acceptance limit.

	IC50 values (µg/ml) of virus stocks determined with HIV-1 neutralizing test reagents				
Pseudovirus	sCD4	IgG1b12	2F5	4E10	TriMab
PVO.4 (Rack No. 6 Pos F7)	11.78	>25.00	>25.00	33.62	5.58
PVO.4 (Rack No. 5 Pos F7)	12.35	>25.00	>25.00	36.04	5.84
PVO.4 (manual reference stock)	9.97	>25.00	>25.00	37.03	5.26
acceptance limit	3.32 to 29.91	>25.00	>25.00	12.34 to 111.09	1.75 to 15.78
