# Supplementary material for: Validation of an automated system for aliquoting of HIV-1 Env-pseudotyped virus stocks
Source: PLoS One. 2018 Jan 4;13(1):e0190669. doi: 10.1371/journal.pone.0190669 (PMC5754138; doi:10.1371/journal.pone.0190669)
Supplement: S17 Table — (PDF) [file pone.0190669.s017.pdf]

S17 Table. Summary of the visual and microscopic evaluation of the sterility tests for (A) the worktable, (B) the system liquid and (C) the virus supply tubing system.

A

Agar plate location	Room temperature	34°C, anaerobic conditions	34°C, aerobic conditions
On worktable	-	-	-
Under worktable (Waste Container)	-	-	-
Virus supply bottle	-	-	-
Negative control	-	-	-
Positive control	+	+	+

B

Agar plates incubated with	Room temperature	34°C, anaerobic conditions	34°C, aerobic conditions
System Liquid	-	-	-
Negative control	-	-	-
Positive control	+	+	+

C

Cell culture flask incubated with	37 °C, aerobic condition
GM without antibiotics pumped through the tubing system	-
Negative control	-
Positive control	+

- no microbial growth was observed
+ microbial growth was observed
